# Blockade of CD73 potentiates radiotherapy antitumor immunity and abscopal effects via STING pathway

**DOI:** 10.1038/s41420-024-02171-4

**Published:** 2024-09-16

**Authors:** Ran An, Chao Wu, Cunyu Tang, Chen Zhang, Feiru Han, Zeen Xu, Yiping Zou, Jun Wang, Zhiyong Yuan, Shengpeng Jiang, Lijie Liu, Chongbiao Huang, Zhen Tao

**Affiliations:** 1 Department of Radiation Oncology, Tianjin Medical University Cancer Institute and Hospital, National Clinical Research Center of Cancer, Key Laboratory of Cancer Prevention and Therapy, Tianjin and Tianjin’s Clinical Research Center for Cancer, Tianjin, PR China; 2 Department of Pancreatic Cancer, Tianjin Medical University Cancer Institute and Hospital, National Clinical Research Center of Cancer, Key Laboratory of Cancer Prevention and Therapy, Tianjin and Tianjin’s Clinical Research Center for Cancer, Tianjin, PR China; 3 Department of Anesthesiology, The First Central Clinical School, Tianjin Medical University, Tianjin, PR China; 4 Department of Oncology, First Hospital of Qinhuangdao, Qinhuangdao, PR China; 5 Department of Oncology, Tongji Hospital, Tongji Medical College, Huazhong University of Science and Technology, Wuhan, Hubei PR China

**Keywords:** Immunology, Cancer

## Abstract

Radiotherapy (RT) is a crucial treatment for colorectal cancer (CRC) patients, but it often fails to induce systemic antitumor immunity. CD73, an immunomodulatory factor, is upregulated after RT and associated with poor prognosis in CRC patients. This study aims to elucidate the mechanisms driving RT-induced CD73 upregulation in CRC and investigate how combining RT with CD73 blockade stimulates immune responses and induces abscopal effects. Findings revealed that RT-induced CD73 upregulation is mediated by the ataxia telangiectasia and Rad3-related (ATR) pathway and correlated with RT tolerance, as demonstrated through flow cytometry, immunofluorescence, and Western Blotting. Using flow cytometry and multicolor immunofluorescence, experiments demonstrated that in CRC subcutaneous tumor models, combination therapy reduces the infiltration of myeloid-derived suppressor cells (MDSCs), tumor-associated macrophages (TAMs), and regulatory T cells (Tregs) while increasing dendritic cells (DCs) and CD8 + T cells, resulting in superior antitumor responses. Additionally, results from flow cytometry, Western Blot, and RNA sequencing demonstrated that combination therapy enhances the antigen-presenting ability of DCs and activates tumor antigen-specific CD8 + T cells, improving their function and delaying their depletion. The activation of the cGAS-STING and IFN-I pathways is crucial for this effect. In summary, the integration of RT with CD73 blockade effectively reverses the immunosuppressive TME and invigorates CD8 + T cell-driven, specific antitumor immune responses. These insights shed fresh light on the mechanisms governing the synergistic modulation of immunity by RT and CD73 blockade in CRC, offering promising avenues for the advancement of therapeutic strategies against CRC.

## Introduction

In recent years, colorectal cancer (CRC) has emerged as a significant global public health challenge, ranking third in terms of incidence and second in cancer-related mortality [1, 2]. It continues to be a leading cause of cancer-related deaths worldwide. Radiotherapy (RT) plays a pivotal role in the treatment of CRC, particularly in the neoadjuvant setting, where it effectively reduces tumor volume prior to surgery, thereby enhancing resectability and potentially leading to a lower rate of local recurrence [3]. The neoadjuvant approach allows for more precise targeting of the tumor and can also facilitate the preservation of organs, such as the rectum, which is particularly relevant for patients with rectal cancer [4]. Moreover, preoperative RT can modulate the tumor microenvironment (TME), enhancing its immunogenicity and potentially priming the immune system for a more effective response against cancer cells.

However, a subset of CRC patients exhibits poor responsiveness to RT, which can be attributed to various factors. Certain tumor types, including CRC, are inherently radioresistant, significantly impacting effective treatment. The effectiveness of RT is further contingent on optimizing the dose, fractionation, and employing advanced techniques to reduce damage to healthy tissues, all critical for maximizing therapeutic outcomes and minimizing toxicity. It is becoming increasingly clear that RT can modulate the expression of numerous immunosuppressive molecules, which may influence the effectiveness of the treatment [5]. For instance, RT has been shown to upregulate the expression of programmed cell death protein 1 ligand 1 (PD-L1) on tumor cells, which can inhibit T-cell activation and proliferation, thereby reducing the efficacy of RT [6]. This upregulation occurs through various mechanisms, such as the activation of the DNA damage repair pathway, which can lead to the stabilization and accumulation of the transcription factor p53, a known inducer of PD-L1 [7]. Moreover, RT has been observed to induce the expression of additional immunosuppressive molecules, including CD73, in preclinical models of various cancers, such as CRC and breast cancer [8, 9]. This upregulation contributes to the establishment of a TME that is less conducive to the function of immune cells, potentially hindering the effectiveness of the immune response against the tumor.

CD73, an enzyme intricately involved in TME regulation, plays a pivotal role in tumor immune evasion [10, 11]. Teaming up with CD39, CD73 engages in a sequential ATP hydrolysis process, leading to adenosine accumulation, thereby fostering tumor proliferation and dampening immune responses [12–14]. Adenosine binds to receptors on both tumor and immune cells, exerting a modulatory effect on antitumor immunity by inhibiting the function of protective immune cells (e.g., effector T cells, NK cells, DCs, and B cells), while maintaining the function of regulatory immune cells (e.g., Tregs, MDSCs, TAMs, and CAFs). This plays a critical role in tumor immune evasion by fostering an immune-tolerant microenvironment that facilitates tumor evasion from the immune system’s surveillance. Furthermore, the CD73-adenosine axis has been implicated in promoting neovascularization, metastasis, and cancer cell survival [15]. A correlation between elevated CD73 expression and poor prognosis has been established in a variety of solid tumors, including breast and ovarian cancers [16, 17]. In the context of CRC, the expression of CD73 has been demonstrated to affect T cell receptor diversity and transcriptional profiles of T cells, thus suggesting their critical roles in T cell exhaustion within tumors [18]. Notably, in various preclinical cancer models, including CRC, the combined application of RT and CD73 blockade has been explored, leveraging the synergistic antitumor immune effects of this dual approach [8, 19, 20]. However, the precise mechanisms by which RT upregulates CD73 expression in CRC, and its correlation with patient response to RT, remain elusive. Furthermore, the intricate immunological underpinnings of the combined therapy’s impact on the immune response necessitate further investigation.

While RT is recognized for its ability to activate the immune system, particularly through the cGAS-STING pathway, which plays a pivotal role in the initiation and amplification of antitumor immune responses [21]. However, as a “double-edged sword” influencing immunotherapy, RT also inhibits immune responses through various mechanisms. The upregulation of CD73 caused by RT poses a significant challenge for the treatment of CRC patients, which may be the reason for the development of resistance to RT therapy in CRC patients. Importantly, our study has uncovered for the first time that simultaneous inhibition of CD73 and RT further activates the cGAS-STING pathway. The addition of CD73 blockade enhances the antitumor immune response of RT, effectively weakening the immunosuppressive effect of RT, thereby allowing RT to become a more precise and potent weapon in activating immunity.

Here, we explored the role of RT in modulating the expression of CD73 in CRC cells through the ataxia telangiectasia and Rad3-related (ATR)-mediated DNA damage repair pathway, a mechanism closely associated with the development of RT tolerance in CRC patients. Notably, our findings in preclinical CRC models demonstrated that the combination of RT with CD73 blockade significantly enhanced the activation of the cGAS-STING pathway, thereby reversing the immunosuppressive TME. This therapeutic approach promoted DC antigen presentation and induced CD8 + T cell-mediated, tumor-antigen-specific immune responses, leading to improved antitumor effects and the occurrence of abscopal effects. These insights shed a fresh light on the mechanisms governing the synergistic modulation of immunity by RT and CD73 blockade in CRC, offer a solid theoretical basis for therapies targeting the RT-regulated CD73 pathway, and providing broad prospects for advancing CRC therapeutic strategies.

## Results

### RT-induced CD73 upregulation impacts RT response in CRC patients

We first identified a significantly higher expression of CD73 in CRC tumor tissues than in normal adjacent tissues by analyzing CD73 expression in the public dataset. (Fig. S1A). Consistent with previous studies linking elevated CD73 levels to poor clinical outcomes in various tumor types, we found that higher CD73 expression in a variety of solid tumors including CRC was associated with shorter overall survival times (Fig. S1B–D), suggesting CD73 may serve as a prognostic marker for CRC patients.

**Fig. 1:**
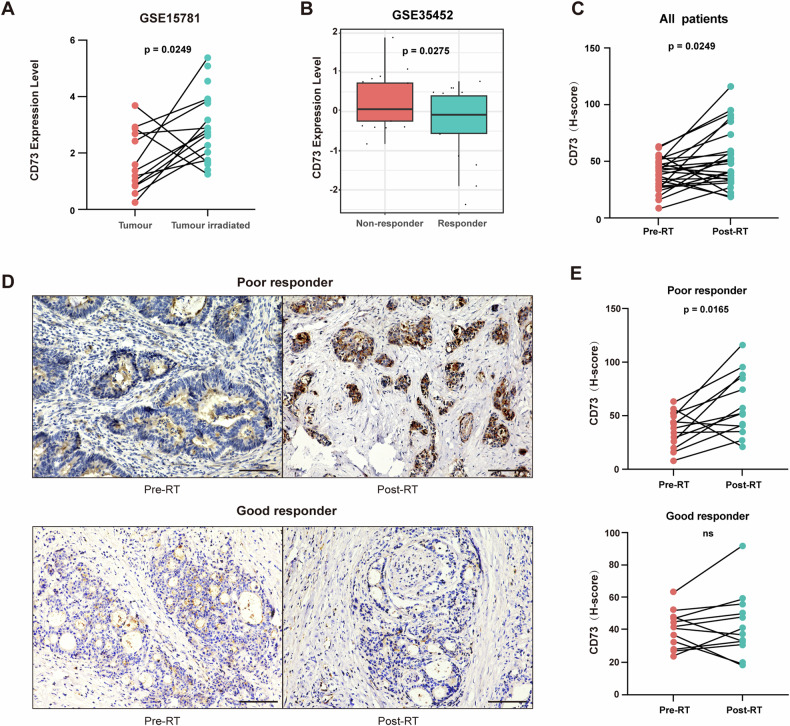
The elevated levels of CD73 induced by RT may serve as a mechanism of immune evasion in CRC patients. **A** The CD73 expression in CRC tumors and paired post-RT CRC cells. **B** The CD73 expression in responders and non-responders to preoperative chemoradiotherapy. H-scores (**C**, **E**) and representative images (**D**) of CD73 IHC surface staining on CRC cells in pretreatment biopsies versus postirradiation surgical specimens in 25 patients with rectal cancer who underwent neoadjuvant RT followed by en bloc resection. Good responders, TRG 0–3; Poor responders, TRG 4 to 5. Scale bars: 100 μm. Statistical differences were examined using the paired Student *t* test.

To investigate the effect of CD73 expression on CRC patients’ response to RT, we compared CD73 mRNA levels and established that RT increases CD73 expression (Fig. 1A). Notably, higher CD73 expression in post-RT samples correlated significantly with poorer RT response (Fig. 1B). To validate our findings, an immunohistochemistry (IHC) study on 25 neoadjuvant RT-treated rectal cancer patients was conducted. CD73 expression before and after irradiation was assessed using histoscores and the extent of changes in tumor tissue after irradiation, indicative of the response to RT, was evaluated using Tumor Regression Grade (TRG). Good responders (TRG 0-3) and poor responders (TRG 4-5) exhibited distinct CD73 expression patterns [22]. Surgical specimens collected post-RT displayed markedly elevated CD73 levels compared to pre-RT paired biopsy samples (Fig. 1C). This increase was particularly pronounced in poor responders, while there was no significant difference in CD73 expression among good responders (Fig. 1D, E and Table. S2), supporting the idea that RT-induced CD73 upregulation was significantly associated with RT tolerance in CRC patients.

### RT up-regulates CD73 in MC38 cells, which depends on the DNA damage repair pathway

**Fig. 2:**
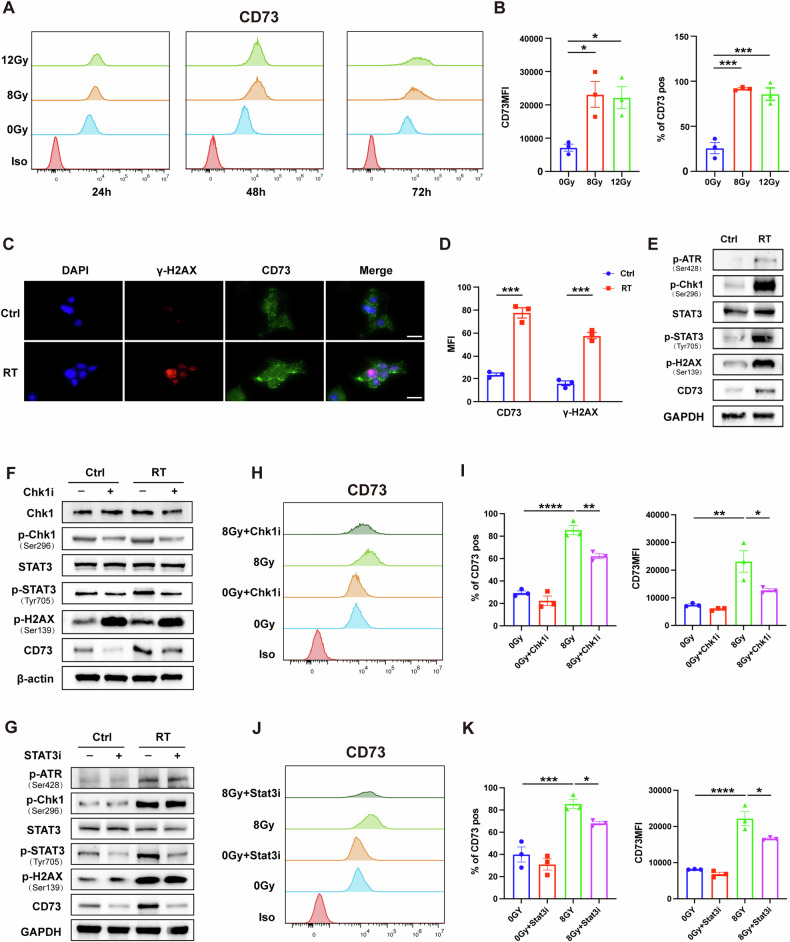
RT up-regulates CD73 in MC38 cells, which depends on the DNA damage repair pathway. Flow cytometric analysis of CD73 expression on MC38 cells at different time points after RT. Images (**A**), representative histograms of percentage and MFI (**B**) of CD73 on MC38 cells 48 h after 8 Gy or 12 Gy irradiation. **C**, **D** Immunofluorescence staining analysis of CD73 and γ-H2AX on MC38 cells 48 h after 8 Gy irradiation. Representative images (**C**) and MFI (**D**) of CD73 and γ-H2AX. Scale bars: 100 μm. Western blot analysis of the expression of CD73 and ATR-Chk1-STAT3 pathway in MC38 cells treated with or without 8 Gy RT (**E**), and ± Chk1i (50 nM, SCH900776) (**F**), ±Stat3i (500 nM, Angoline) (**G**), and harvested 24 h later. Flow cytometric analysis of CD73 expression on the surface of MC38 cells treated ± 8 Gy RT, ±Chk1i (**H**), ±Stat3i (**J**). Percentage and MFI (**I**, **K**) of CD73 on MC38 cells 48 h after RT. Statistical variations were analyzed utilizing the unpaired *t*-test. Data are expressed as mean ± SEM (*n* = 3 per group).*P < 0.05; **P < 0.01; ***P < 0.001; ****P < 0.0001.

To dissect the mechanism behind RT-induced CD73 upregulation, we conducted flow cytometry on MC38 cells pre- and post-RT, with doses of 8 Gy or 12 Gy, assessed at 24 h, 48 h, and 72 h. We found that CD73 expression on tumor cells significantly increased at all three-time points, regardless of radiation dose (Fig. 2A, B). RT induces DNA damage, particularly DNA double-strand breaks (DSBs), necessitating a complex DNA damage repair system in cancer cells. Recent studies have identified that post-irradiation activation of the ATR-mediated DNA double-strand break repair pathway (ATR-Chk1 pathway) stimulates immune checkpoints such as PD-L1 and CD47 via signal transducer and activator of transcription 1/3 (STAT1/3) signaling [22]. STAT3, a key transcription factor, drives the expression of multiple immune checkpoints. Our Immunofluorescence Staining (Fig. 2C, D) and Western Blot (Figs. 2E, S6A, and S7A) results support our hypothesis that the expression of CD73 in MC38 cells is up-regulated by RT through the ATR-Chk1-STAT3 pathway. To validate this mechanism, we used Chk1 and STAT3 inhibitors. Consistent with previous findings, inhibition of the DNA damage repair pathway effectively suppressed CD73 upregulation induced by RT (Figs. 2F–K, S6B, C, and S7B, C). These results collectively demonstrate that the upregulation of CD73 expression following RT in CRC is intricately linked to the induction of the DNA damage repair pathway.

### CD73 blockade enhanced RT-mediated specific antitumor immune effects and remodeled the TME

**Fig. 3:**
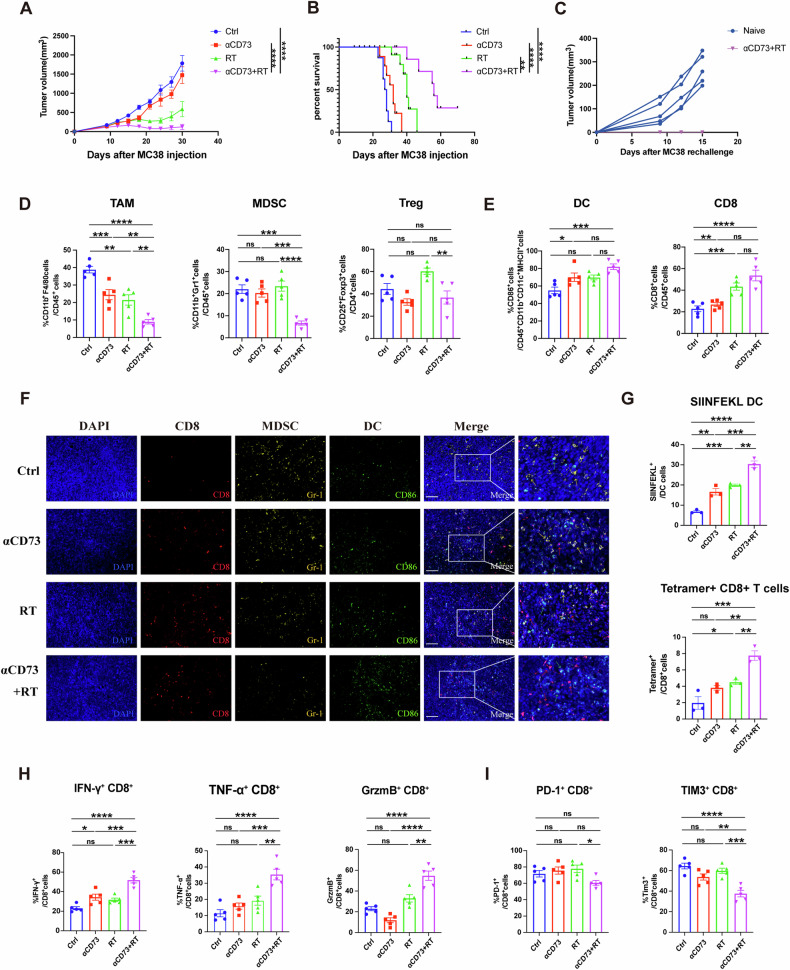
CD73 blockade enhanced RT-mediated antitumor immune effects and remodeled the TME. C57BL/6 mice were given through s.c. injection into the right flank with 1 × 10^6^ MC38 cells. Once tumor size achieved 100 mm^3^ (about 10 days after injection), a single dose of 8 Gy RT was administered locally to mice. Simultaneously, αCD73 treatment was initiated, with five doses administered every other day. **A** Tumor growth curve of each treatment group. **B** Corresponding survival data of each treatment group. **C** Tumor growth curves of naïve C57BL/6 mice or completely responsive mice from previously treated combination (RT + αCD73) group rechallenged with MC38 cells. **D**, **E** Ten days after RT, immune cell populations in the TME were analyzed using flow cytometry. Quantification of TAMs (CD11b + F4/80+), and MDSCs (CD11b+Gr-1+) as a proportion of live CD45+ cells in the tumor, Tregs as a proportion of live CD4+ cells in the tumor (**D**). Quantification of mature DCs (CD86+) as a proportion of CD11b + CD11c+MHC-II+ cells in the tumor, CD8 + T cells as a proportion of live CD45+ cells in the tumor (**E**). **F** Multispectral immunofluorescence imaging reveals CD8+, Gr1+, and CD86+ cell infiltration in tumors. Scale bars: 20 μm. **G** Quantification of H-2KbSIINFEKL+ cross-presenting DCs (SIINFEKL+) as a proportion of CD11b + CD11c+ cells, and H2-Kb - SIINFEKL-tetramer+ CD8 T+cells (Tetramer+) as a proportion of CD8+ cells in the tumor. **H** Quantification of IFN-γ+, TNF-α+, and GrzmB+cells as a proportion of CD8+ cells in the tumor. **I** Quantification of PD-1+ and TIM3+ cells as a proportion of CD8+ cells in the tumor. Statistical variations were analyzed utilizing the One-way ANOVA (**A**, **D**, **E**, **G**, **H**, **I**) or Kaplan–Meier method with the log-rank test (**B**). Data are expressed as mean ± SEM (*n* = 10 per group).*P < 0.05; **P < 0.01; ***P < 0.001; ****P < 0.0001.

To validate our hypothesis that RT combined with CD73 blockade could enhance antitumor responses, we implanted MC38 (1×10^6^) cells on C57BL/6 mice’ right flanks. After ten days, we administered an 8-Gy irradiation to the tumors. Simultaneously, we initiated αCD73 treatment, consisting of five doses every other day, with group assignments based on initial tumor volumes (Fig. S2A). Anti-CD73 antibodies alone minimally affected MC38 tumor growth or overall survival. RT alone temporarily controlled tumors and improved survival compared to the untreated group. However, combination treatment significantly suppressed tumor growth and enhanced survival compared to the untreated and monotherapy groups (Figs. 3A, B and S2B). Three previously cured mice and five naïve mice underwent re-challenge with MC38 cells. Notably, tumors grew rapidly in the naïve mice but failed to establish in the completely responsive mice even 15 days post-rechallenge (Fig. 3C). This data underscores the combination of local RT and αCD73 enhances tumor radiosensitivity and fosters an antitumor immune memory response.

To further investigate how the combination treatment alters the TME, we still utilized the MC38 tumor models and analyzed the immune microenvironment via flow cytometry. The results demonstrated a significant reduction in the infiltration of TAMs (CD45 + CD11b + F4/80+), MDSCs (CD45 + CD11b+ Gr-1+), and Tregs (CD4 + CD25+ Foxp3+) in the combination treatment group (Figs. 3D and S3A). While the RT treatment group showed a slight increase in MDSCs and Tregs, this was reversed with the addition of an anti-CD73 antibody. Furthermore, we observed that either RT or CD73 blockade alone enhanced the infiltration of mature DCs (CD11b + CD11c+ MHC-II + CD86+) and CD8 + T cells, but the combination treatment led to a more significant enhancement (Figs. 3E and S3B). Immunofluorescence staining confirmed these findings, showing consistent distribution patterns of immune cells, including MDSCs, DCs, and CD8 + T cells within the tumors (Figs. 3F and S3C). Moreover, there was a notable increase in the expression of OVA-derived SIINFEKL peptide on H-2Kb complexes on DCs. Simultaneously, the generation of H-2Kb-SIINFEKL tetramer-positive CD8 T cells also increased, as demonstrated in experiments involving the implantation of MC38-OVA tumor cells (Figs. 3G and S3D). In addition, we discovered an increased percentage of CD8 + IFN-γ+ cells, CD8+ granzyme B+ (GzmB) cells, and CD8 + TNF-α+ cells in tumors receiving RT + αCD73 combination treatment (Figs. 3H and S3E). Meanwhile, there was a significant decrease in the expression of PD-1 and TIM-3 compared to monotherapy (Figs. 3I and S3F). Altogether, these findings revealed that RT combined with CD73 blockade effectively reversed the immunosuppressive microenvironment in MC38 tumors. It activated antigen presentation in DCs, enhanced the priming of tumor antigen-specific CD8 + T cells, improved CD8 + T cell function, and delayed their exhaustion, likely contributing to the robust antitumor response observed with this combination therapy.

### CD8 is indispensable in blocking CD73 to enhance RT-mediated immune responses and abscopal effects

**Fig. 4:**
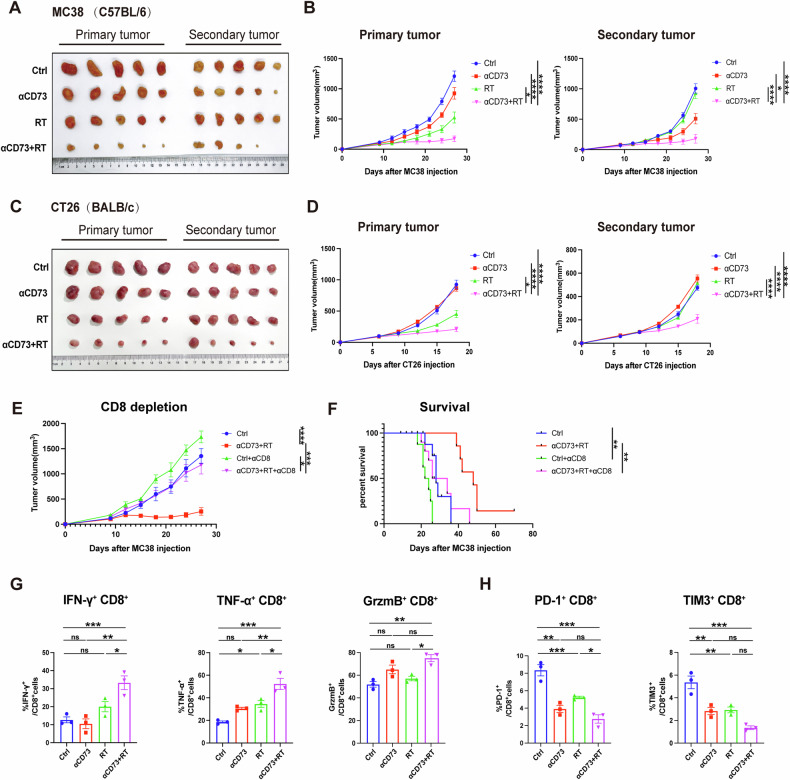
CD8 is indispensable in blocking CD73 to enhance RT-mediated systematic immune responses and abscopal effects. 1 × 106 MC38 cells were injected into the left flank of C57BL/6 mice three days after the first injection (1 × 106) into the right flank. For CT26 model, we utilized 2 × 105 CT26 cells injecting into BALB/c mice. The pictures of primary and secondary tumors of C57BL/6 (**A**) and BALB/c mice (**C**), and their tumor growth curves of each treatment group (**B**, **D**) are shown. **E**, **F** In CD8 + T cells depletion experiment, the first anti-CD8 antibodies were administered three days prior to RT, with subsequent applications twice weekly throughout the course of treatment. Tumor growth curves of each treatment group (**E**), with corresponding survival data (**F**), are shown. **G**, **H** Splenic CD8 + T cells were isolated using magnetic bead isolation (Miltenyi Biotec) and pre-activated with Dynabeads mouse CD3/CD28 beads (IBA Life Sciences) for 48 h. After 48 h of pre-activation, they were co-cultured with MC38 cells treated ± 8 Gy RT, ±CD73i for 48 h, followed by flow analysis 24 h later. **G** Quantification of IFN-γ+, TNF-α+, and GrzmB+ cells as a proportion of CD8+ cells. **H** Quantification of PD-1+ and TIM3+ cells as a proportion of CD8+ cells. Statistical variations were analyzed utilizing the One-way ANOVA (**B**, **D**, **E**, **G**, **H**) or Kaplan–Meier method with the log-rank test (**F**). Data are expressed as mean ± SEM (*n* = 10 per group).*P < 0.05; **P < 0.01; ***P < 0.001; ****P < 0.0001.

To assess the potential of combining αCD73 with RT for inducing abscopal effects on non-irradiated tumors, we implanted MC38 cells on both the right (primary, irradiated) and left (secondary, non-irradiated) sides of mice and administered various treatment regimens (Fig. S4A). The combination therapy group exhibited slower growth of non-irradiated tumors compared to any single treatment (Fig. 4A, B). We next repeated the experiment using another CRC cell CT26 (2 × 10^5^) in BALB/c mice, and we observed similar results (Fig. 4C, D), indicating that CD73 blockade enhances RT’s effectiveness on primary tumors and stimulates systemic abscopal effects in CRC.

Recognizing CD8 + T cells’ pivotal role in tumor immune responses, we tested whether T cell responses are crucial for the antitumor efficacy of the combination therapy using anti-CD8 antibodies in MC38 mice. Depleting CD8 + T cells compromised the survival outcomes of the combination treatment and distinctly abrogated its effect on tumor growth (Figs. 4E, F, and S4B), emphasizing the essential contribution of CD8 + T cells to the effectiveness of the combination therapy.

Then we extracted CD8 + T cells from the spleen of C57BL/6 mice. After co-culturing the cells with MC38 cells with or without 8-Gy RT and anti-CD73 antibody treatment for two days, we measured the proportion of effector CD8 + T cells and the expression levels of CD8 surface immune checkpoints (Fig. S4C). As expected, flow cytometry analysis showed that the combination therapy also significantly enhanced CD8 + T cell function and delayed CD8 + T cell exhaustion in vitro (Figs. 4G, H and S4D, E).

### The combination of CD73 blockade and RT improves T-cell function which relies on the STING pathway

**Fig. 5:**
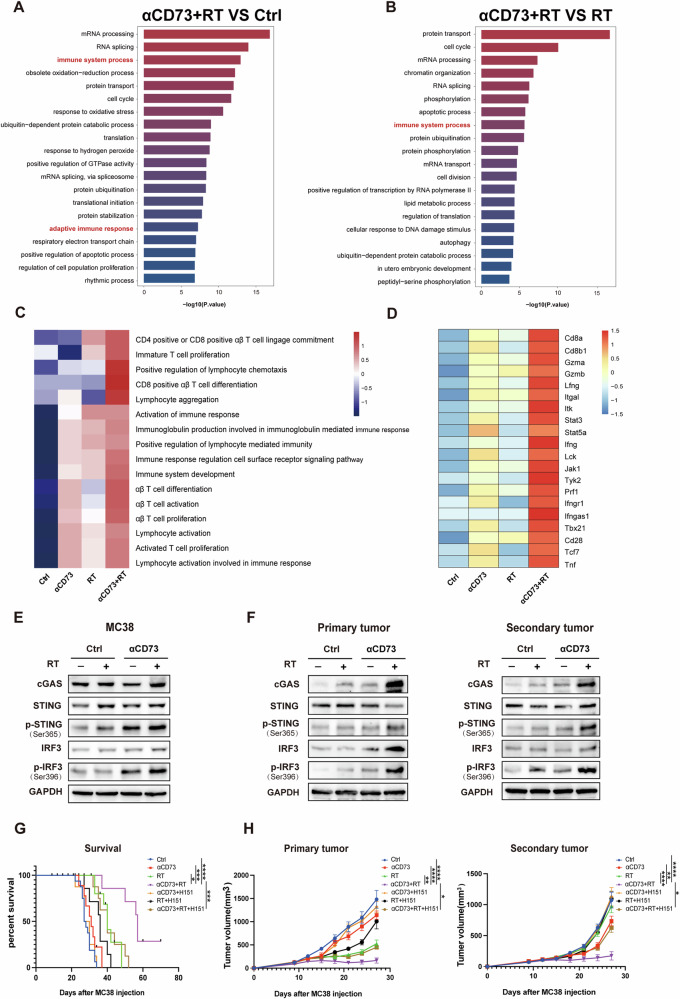
The combination of CD73 blockade and RT remodels the TME which relies on the STING pathway. **A** The top 20 GO BP enrichment pathways in the αCD73 + RT group (αCD73 + RT group vs control group), (**B**) and in the αCD73 + RT group (αCD73 + RT group vs αCD73 group). **C** GSVA analyses of T cell activation–associated pathways of four different groups. **D** DEG analyses in genes associated with CD8 function and the I-IFN pathway in TILs across different treatment groups. E Western blot analysis of the expression of cGAS, STING, pSTING, IRF3, pIRF3, and GAPDH on MC38 cells treated ± 8 Gy RT, ± CD73i, (**F**) and on MC38 cells extracted from both sides of mice treated ± 8 Gy RT, ±CD73i. **G**, **H** In STING inhibition experiment, H151 was administered three days prior to RT, with subsequent applications twice weekly throughout the course of treatment. Tumor growth curves of each treatment group in primary sites and abscopal sites (**H**), with corresponding survival data (**G**) are shown. Statistical variations were analyzed utilizing the One-way ANOVA (**H**) or Kaplan–Meier method with the log-rank test (**G**). Data are expressed as mean ± SEM (*n* = 10 per group).*P < 0.05; **P < 0.01; ***P < 0.001; ****P < 0.0001.

To explore the mechanisms through which T lymphocytes facilitate potent innate and adaptive immune responses in combination therapy, we isolated tumor-infiltrating lymphocytes (TILs) from tumors following 8 days of RT. SMART-Seq (PRJNA1005920) analyses were conducted to compare alterations in gene expression among the various treatment groups (Fig. S4A). Through a comparative analysis of differentially expressed genes and Gene Ontology (GO) analysis, we observed the activation of immune-related pathways in the combination treatment group when compared to both the control group and the RT group (Figs. 5A, B and S4B, C). Gene set variation analysis (GSVA) further revealed significant activation of several T-cell activation-related pathways in response to the combination treatment (Fig. 5C). Additionally, we noted significant upregulation of genes associated with CD8 + T cells function and the I-IFN pathway in the combination treatment group (Fig. 5D).

RT is known to promote DC maturation and I-IFN release, which activates CD8 + T cell-mediated adaptive immune responses via the cGAS-STING pathway [21, 23]. The combination of RT with STING agonizts has been demonstrated to synergistically enhance immune responses in the treatment of various types of tumors [24, 25]. A recent study also discovered that CD73 inhibitors activate the STING pathway in a pancreatic cancer model [26]. Based on these findings, we hypothesized that combining CD73 blockade with RT would lead to more effective antitumor immunity by further enhancing cGAS-STING activation.

To test this hypothesis, Western Blot experiments confirmed increased cGAS expression, STING activation, and downstream IFN regulatory factor 3 (IRF3) phosphorylation in MC38 cells treated with RT and anti-CD73 antibodies (Figs. 5E, S6D, and S7D). Similarly, Western blot analysis of subcutaneous tumor tissue from mice receiving various treatments yielded the same results (Figs. 5F, S6E, F, and S7E, F). The results supported our conjecture, providing further evidence of the enhanced cGAS-STING activation with the combination therapy. Additionally, we conducted in vivo validation using the STING inhibitor H151. Notably, the use of this inhibitor significantly compromised the effectiveness of combination therapy, leading to accelerated tumor growth and reduced mouse survival in the combination group (Fig. 5G, H). These findings strongly indicate that combination therapy fosters a potent CD8-mediated antitumor immune response by boosting the cGAS-STING innate immune sensor circuit and subsequent type I IFN production.

## Discussion

**Fig. 6:**
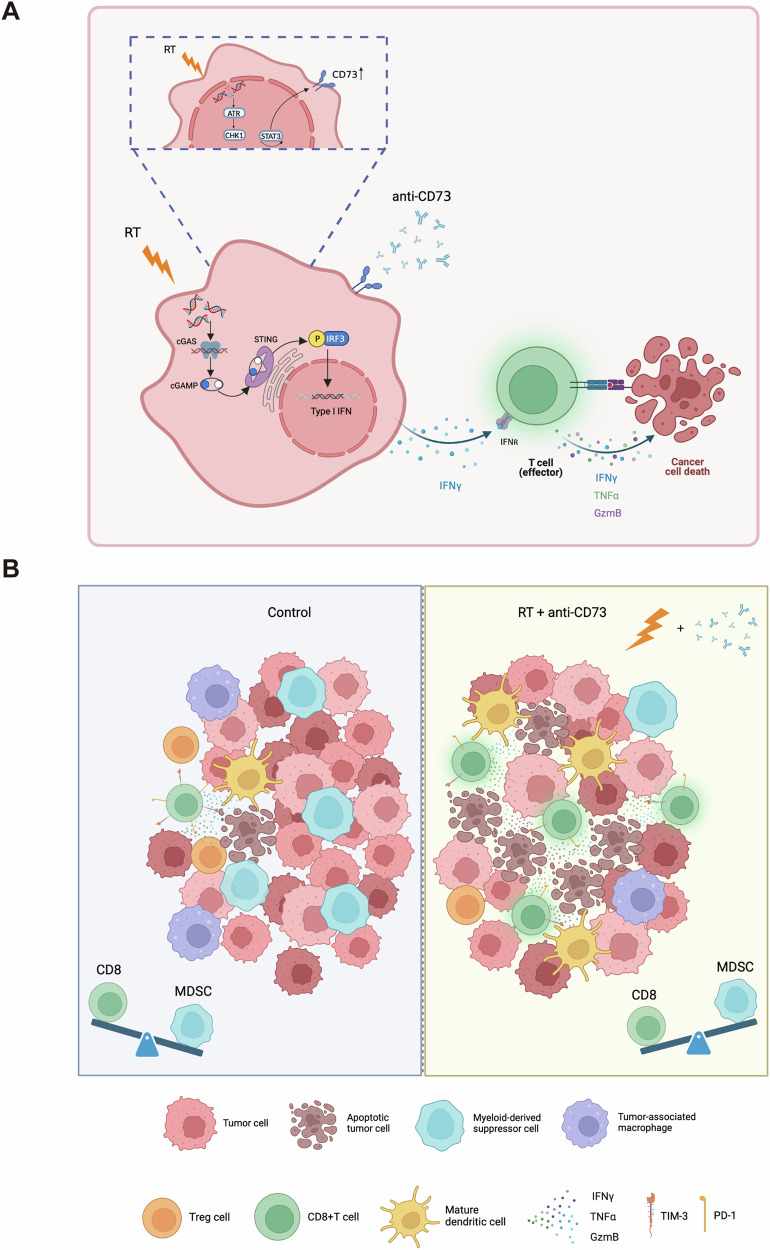
A mechanistic roadmap explaining how RT upregulates CD73 and how combination therapy exerts antitumor effects. **A** Schematic diagram of RT up-regulates CD73 in MC38 cells through ATR-Chk1-STAT3 pathway and the combination of αCD73 and RT triggers CD8 + T cell-mediated adaptive immune responses by potentiating activation of the cGAS-STING pathway and type I IFN production. **B** The schematic summary of synergistic mechanism between αCD73 + RT. The combination of αCD73 and RT (right) significantly reduces the infiltration of TAM, MDSCs, and Tregs, while augmenting the infiltration of mature DCs and CD8 + T cells. Furthermore, it stimulates the generation of TNF-α, IFN-γ, and GzmB and reduces T-cell exhaustion compared to the control group (left).

In this study, we investigated the impact and underlying mechanism of CD73 blockade on RT-induced antitumor immune responses in CRC (Fig. 6A, B). Our results demonstrated that RT upregulates the expression of CD73 through the ATR-mediated DNA damage repair pathway in CRC. The combination of RT and αCD73 effectively remodels the TME of CRC by reversing the infiltration of immunosuppressive cells, enhancing antigen presentation by DCs, and augmenting the cytotoxic function of CD8 + T cells while delaying their exhaustion. Additionally, the combined therapy induced a pronounced abscopal effect, suggesting its potential to elicit systemic effects on tumors outside the irradiated field in mouse models of CRC. Furthermore, RT with the blockade of CD73 led to the activation of cellular cGAS-STING and IFN-I pathways, which played a critical role in initiating tumor-specific immune responses mediated by CD8 + T cells.

RT can induce DNA damage, directly or indirectly activating the ATR-Chk1 pathway [27–29]. ATR plays a pivotal role in error-free HR DNA double-strand break repair and replication stress response. Activation of this pathway triggers a cascade of signaling events, including cell cycle arrest, initiation of DNA repair, and alterations in gene expression related to immunity [30–32]. Furthermore, post-irradiation activation of STAT3 contributes to immune suppression and radiation resistance in various tumor models [33, 34]. Therefore, we have successfully established a significant and intricate connection between the upregulation of CD73 on CRC cells following RT and the DNA damage repair pathway. In summary, the upregulation of CD73 in CRC cells by RT relies on the ATR-Chk1-STAT3 pathway, which is significantly correlated with radiotherapy tolerance in CRC patients.

RT is a critical tool in cancer treatment, recognized for its ability to induce direct cytotoxic effects on tumor cells through the generation of DNA double-strand breaks [27]. But its impact on immunotherapy is often described as a double-edged sword due to its complex and multifaceted effects on the TME and the immune system. On the beneficial side, RT can stimulate an antitumor immune response by promoting the release of tumor-associated antigens from dying cancer cells, a process known as immunogenic cell death. This can lead to the activation of dendritic cells and the subsequent priming of T lymphocytes, setting the stage for an adaptive immune response against the tumor [35]. Additionally, RT can activate the cGAS-STING pathway within tumor cells, leading to the production of IFN-I, which can further enhance the immune response against the tumor [23, 36]. However, RT can also have immunosuppressive effects that may limit its therapeutic potential. RT can stimulate the mobilization and expansion of immunosuppressive cells within the TME. These include regulatory Tregs, MDSCs, and TAMs, which can dampen the antitumor immune response. Moreover, RT can induce a state of chronic inflammation within the TME, leading to the activation of immunosuppressive pathways and the recruitment of additional immunosuppressive cells. This can result in a more hostile environment for immune cells and contribute to the development of radioresistance [37, 38]. Additionally, RT can modulate the expression of numerous immunosuppressive molecules. Except PD-L1, RT can also induce the expression of indoleamine 2,3-dioxygenase (IDO) and adenosine, which contribute to the creation of a hostile environment for immune cells [39–41]. Adenosine, a metabolite that accumulates in the TME, significantly contributes to immunosuppression by binding to its receptors (A1, A2A, A2B, and A3) on immune cells. This action can dampen T-cell responses and bolster the activity of Tregs, thus fostering a TME that is less conducive to effective immunotherapy [42]. The ectoenzymes CD39 and CD73 play pivotal roles in this process by catalyzing the production of adenosine, highlighting them as potential therapeutic targets.

Current research is abundant with studies exploring the targeting of the adenosine pathway, particularly in combination with immune checkpoint inhibitors (ICI), RT, and chemotherapy. This approach has shown promise in enhancing treatment efficacy, and numerous clinical trials are now underway to evaluate these combined strategies. In mouse models of CRC, the combination of CD73 inhibition with RT has been shown to activate immune responses synergistically, leading to effective suppression of tumor growth [9, 19]. It’s worth noting that comparable results have been observed in models of breast and pancreatic cancer, where the combination of CD73 targeting with RT, further augmented with CTLA-4 and PD-1 immune checkpoint inhibitors, respectively, demonstrated enhanced antitumor activity [8, 20]. In glioblastoma model, the combination of CD73 deficiency with RT and anti-CTLA-4 and anti-PD-1 therapies has also exhibited synergistic effects [43]. The success of these combinatorial approaches in various cancer models underscores the potential for similar strategies to be effective in CRC. Additionally, the co-inhibition of CD73 and A2AR adenosine signaling improves antitumor immune responses, offering another layer of potential therapeutic intervention [44]. In the context of CRC, such multiple targeting in combination with RT could provide a novel synergistic treatment strategy, further enhancing the immune response against tumors. The integration of RT with CD73 blockade and additional potential targets in CRC represents a promising direction for our future research endeavors.

While the synergistic immunostimulatory effects of combining RT with CD73 blockade have been established across various cancer models, the exploration of its mechanism has been rarely reported. Wennerberg et al. showed that combining CD73 blockade with RT boosted cDC1 tumor infiltration and systemically enhanced antitumor T-cell responses in the TS/A model, independent of IFN [8]. Similarly, Meziani et al. found that the antitumor efficacy of CD73 blockade with RT in the MC38 model was associated with changes in iCOS expression on CD4 + T cells [19]. Expanding upon these findings, our study sought to explore additional mechanisms of action underlying the combination of RT and CD73 blockade. Our investigations confirmed the pivotal role of CD8 + T cells in mediating the antitumor immune response triggered by this combination therapy. Notably, we observed activation of the cGAS-STING pathway upon combination treatment, subsequently activating the function of CD8 + T cells and resulting in a robust, tumor-specific immune response and the induction of abscopal effects. This discovery suggests that the activation of the cGAS-STING pathway may serve as a crucial molecular mechanism driving the immunological effects of the combined treatment. Moreover, our results demonstrated that inhibition of STING significantly attenuated the antitumor efficacy of the combination therapy, underscoring the critical role of this pathway in mediating the immune response. However, we did not explore the potential synergies of combining STING agonizts with RT and CD73 blockade, representing an important avenue for our future research. Investigating such synergistic effects could yield valuable insights into enhancing the therapeutic potential of this combination treatment for CRC patients.

Beyond the current findings, it is important to acknowledge the limitations inherent in our study. The small sample size of 25 cases may limit the robustness of our results. Additionally, the reliance on a single type of animal model, without corroboration from in situ, metastatic, orthotopic, or synthetic models, could affect the reliability of our assessments. Our future research will aim to expand the sample size with additional case inclusions and will incorporate a broader range of animal models to enhance the validation of our findings, thereby strengthening the evidence base for our conclusions.

Overall, our study is the first to reveal that RT upregulates CD73 expression through the ATR-mediated DNA damage repair pathway and validates this pathway as a cause of radiotherapy tolerance in CRC patients. We have discovered a novel mechanism by which RT combined with CD73 blockade activates immunity in CRC models. This involves further activation of the cGAS-STING pathway and the generation of IFN-I, which promotes DCs antigen presentation and initiates CD8 + T cell-mediated tumor antigen-specific immune responses. Our findings offer novel perspectives on the immunological mechanisms by which RT in combination with CD73 blockade modulates the treatment of CRC. It also underscores the necessity for further research to fully harness the potential of these therapies in CRC treatment.

## Materials and methods

### Cell lines, animals, and reagents

The mouse CRC cell lines MC38 and CT26 were purchased from BMCR (http://www.cellresource.cn/). Cells were cultivated in RPMI-1640 enriched using 10% fetal bovine serum (FBS), 10 mg/mL penicillin-streptomycin, and 0.1 mmol/L nonessential amino acids (all materials from Gibco) at 37 °C in a humidified incubator containing 5% CO_2_.

Female C57BL/6J mice and BALB/c mice (age, 6–8 weeks) were purchased through SPF (Beijing) Biotechnology Co., Ltd. Food and water were available to all mice at all times under pathogen-free conditions.

Anti-mouse CD73 antibody (clone TY/23), and anti-mouse CD8 antibody (clone YTS 169.4), were acquired through Bio X Cell. Anti-mouse STING antibodies (clone H151) were acquired through Selleck.

### Clinical samples and Immunohistochemical staining

The pretreatment biopsy and post-RT surgical specimens were retrospectively collected from 25 CRC patients in 2022. Paraffin-embedded sections were first incubated with anti-CD73 (1:200 dilution, Abcam, ab175396), followed by goat anti-rabbit secondary antibodies conjugated with horseradish peroxidase. A 2-Solution DAB Kit (Invitrogen) was used to visualize antibody binding. The intensity of staining was graded into negative (0), low (1+), medium (2+), and high (3+), and the histoscore was calculated using the following formula: [1 × (% cells 1+) + 2 × (% cells 2+) + 3 × (% cells 3+)] [22].

### In vivo tumor growth experiments

For this study, unilateral or bilateral subcutaneous tumor models were established. 1 × 10^6^ MC38 cells or 2 × 10^6^ MC38-OVA cells were injected subcutaneously on the right flank of C57BL/6J mice (*n* = 10 per group) and 2 × 10^5^ CT26 cells were injected subcutaneously on the right flank of BALB/c mice (*n* = 9 per group) on day 0, with or without on the left flanks for assessment of abscopal responses on day 3. Once primary tumors reached ~100 mm^3^ volume (on Day 10), the mice were treated with either anti-CD73 antibody (10 mg/kg) by intraperitoneal injection (i.p.), RT of 8 Gy on primary tumors, or the combination. For tumor rechallenge experiments, 1 × 10^6^ MC38 tumor cells were implanted on the right flanks (ipsilateral flank) of the wild-type mice, while an equivalent number of cells (1 × 10^6^) were implanted on the left flank (contralateral flank) of completely response mice 40 days after their primary challenges. For the CD8 + T-cell depletion tests and the STING inhibition tests, antibodies were administered 3 days before RT and twice weekly for the duration of the experiment. Tumor growth and survival were assessed every 3 days using electronic calipers to measure the length and width and calculated by L × W^2^/2.

### Flow cytometry

Tumor tissues were cut up into small pieces 10 days after RT and then digested at 37 °C for 60 min with 1 mg/mL collagenase IV (Sigma-Aldrich) and 0.2 mg/mL DNaseI (Life Technologies) to produce single-cell suspensions. Dissociated cells were washed three times in phosphate buffer saline (PBS) with 5% FBS before being passed through a 70-M cell strainer twice. To achieve single-cell suspension, spleens from tumor-bearing animals were homogenized and passed through a 70-M cell strainer in ice-cold PBS with 5% FBS. ACK Lysis Buffer was used to lyse red blood cells (Life Technologies). For intracellular cytokine staining, cells were stimulated with 50 ng/ml PMA, 500 ng/ml ionomycin, and 10 µg/ml GolgiPlug (BD Bioscience) at 37 °C for 4 h. Cells were then harvested, fixed, and permeabilized using cytofix/cytoperm fixation/permeabilization kit (BD Biosciences). Cells were stained with the following antibodies obtained from BioLegend and eBioscience (Table. S1).

### Multiplexed immunofluorescence staining

Multiple immunofluorescence staining was performed using a PerkinElmer Opal 7-color Technology Kit (NEL81001KT). Sections of the tumor samples from the paraffin-embedded blocks were cut at a thickness of 4 mm. The sections were rehydrated in ethanol after being deparaffinized in xylene. EDTA buffer (PH = 9.0) was used to perform microwave repair for 20 min. The tissue was sealed with an antibody blocker at room temperature after cooling. The sections were then incubated overnight in a refrigerator at 4 °C with the primary antibody before being co-incubated with poly-HRP-MS/Rb for 10 min at room temperature on the second day. Opal TSA was used for visualization (1:100). All samples were stained with the primary antibody for CD8 (catalog No. 98941, CST) and visualized with Opal520 TSA, CD86 (catalog No. 19589, CST) visualized with Opal570 TSA, Gr-1 (clone RB6-8C5,eBioscience) visualized with Opal690 TSA. Finally, the sections were covered with DAPI Fluoromount-G™(Southern Biotech) and cover glass.

Cells were cultured on covered glass in a 24-well plate for 48 h. Following PBS rinsing, cells were fixed with 4% formaldehyde in PBS for 10 min and permeabilized with 0.5% Triton-X100 in PBS for 5 min. After three PBS washes, cells were blocked with 3% BSA in PBS for 1 h, followed by another three PBS washes. Cells were then incubated overnight at 4 °C with the antibody CD73 (catalog No. 32299, SANTA) and H2AX (catalog No. 60566, CST). After three PBS washes, cells were exposed to the corresponding secondary antibodies (ThermoFisher, USA) in 3% BSA, in the dark, at room temperature for 1 h. Following three PBS washes, cells were covered with DAPI Fluoromount-G™(Southern Biotech) and cover glass.

### Switching mechanism at 5′ end of the RNA transcript-sequencing (SMART-seq)

8 days after tumor irradiation, single-cell suspensions (prepared as described above) were obtained. We pooled tumor-infiltrating leukocytes from 10 tumors for each group to increase RNA yields and sample representativeness. Tumor-infiltrating lymphocytes (TILs) were sorted via flow cytometry using BD FACSAria III.

cDNA was fragmented by dsDNA Fragmentase (NEB, M0348S) by incubating at 37 °C for 30 min. Library construction begins with fragmented cDNA. Blunt-end DNA fragments are generated using a combination of fill-in reactions and exonuclease activity, and size selection is performed with provided sample purification beads. An A-base is then added to the blunt ends of each strand, indexed Y adapters are ligated to the fragments, and the ligated products are amplified with polymerase chain reaction. And then we performed the paired-end sequencing on an Illumina NovaseqTM 6000 at the (LC Sciences, USA) following the vendor’s recommended protocol.

### Immunoblotting

MC38 cells were collected and lysed for 30 min on ice in sodium dodecyl sulfate (SDS) lysis buffer supplemented with 1 protease (Sigma-Aldrich, USA) and 1 phosphatase (Bimake, USA) inhibitor before being centrifuged at full speed for 10 min at 4 °C. The supernatants were collected and SDS-PAGE was performed, followed by immunoblotting. DNA Damage Antibody Sampler Kit (catalog No. 9947, CST), Mouse-Reactive STING Pathway Antibody Sample (catalog No. 16029, CST), Phospho-Chk1 (Ser296) Rabbit mAb (catalog No. 90178, CST), Chk1 Rabbit mAb (catalog No. 37010, CST), Phospho-Stat3 (Tyr705) Rabbit mAb (catalog No. 9145, CST), Stat3 Rabbit mAb (catalog No. 30835, CST), β-Actin Antibody (catalog No. 4967, CST) and GAPDH Rabbit mAb (catalog No. 92310, CST) were used for immunoblotting.

### In vitro T-cell experiments

Splenic CD8 + T cells were isolated using magnetic bead isolation (Miltenyi Biotec) and preactivated with Dynabeads Mouse CD3/CD28 beads (IBA Life Sciences) for 48 h, which were cultured in RPMI 1640 with 10% FBS, 10% MEM NEAA (Gibco), 10% GlutaMAX™(Gibco), 10% Sodium Pyruvate (Gibco), 10% 1 M HEPES (Solarbio), 1% 2-Mercaptoethanol (Gibco), and 1uM IL-2 (Pepro Tech). MC38 cells were irradiated with 8 or 0 Gy in culturing medium. At 48 h after RT, CD8 + T cells were co-cultured with MC38 cells with a 10:1 ratio for 24 h adding anti-CD73 or not, then evaluated by flow cytometry.

### Public CRC data

The Cancer Genome Atlas (TCGA) data were obtained from the UCSC Xena Public Data Center. The datasets GSE221575, GSE198758, GSE15781, GSE39582, and GSE35452, including gene expression data and patient clinical information, were downloaded from the Gene Expression Omnibus (GEO).

### Bioinformatic analysis

In the analysis of the public CRC dataset, quality control, dimensionality reduction, clustering, and cellular annotation steps were performed in strict accordance with the parameters provided by the respective studies for single-cell RNA sequencing (sc-RNAseq) data analysis. RNAseq data were transformed into transcripts per million (TPM) to maintain consistency with the microarray results. In the training cohort, patients were categorized into high and low-expression groups based on the median level of CD73 mRNA expression. In addition, Kaplan–Meier survival analysis was used to determine the prognosis of CRC patients.

For SMART-seq data analysis, resulting expression differences were visualized through heatmaps and volcano plots generated using the “pheatmap” package. Differential gene expression (DEG) analysis was performed on the combined mRNA expression data using the “limma” software package, and a threshold for adjusting *p*-values was set. Sample enrichment analysis was conducted using the ‘GSVA’ package. The Fisher test was employed to compute significant enrichments of Gene Ontology (GO) terms and Kyoto Encyclopedia of Genes and Genomes (KEGG) pathway sets within the target gene list.

### Statistical analysis

All findings were recovered from more than two independent experiments. Results are denoted as the mean ± standard error of the mean (SEM) for all figure panels. Survival graphs were analyzed using the Kaplan–Meier method and compared using the log-rank tests. The statistical tests used to determine significant differences in variables between groups, such as Student’s *t*-test, Mann–Whitney U test, or ANOVA, are indicated in the legend. Significant differences in variables between groups were indicated in the figure legends. *P* < 0.05 was considered to be statistically significant. All statistical analyses were performed using the GraphPad Prism software (v9.0.2; La Jolla, CA, USA).

## Supplementary information

supplemental material

Western blot

## Data Availability

The datasets used and analyzed in this study are available from the corresponding author on reasonable request.
