# Supplementary material for: Blockade of CD73 potentiates radiotherapy antitumor immunity and abscopal effects via STING pathway
Source: Cell Death Discov. 2024 Sep 16;10:404. doi: 10.1038/s41420-024-02171-4 (PMC11405876; doi:10.1038/s41420-024-02171-4)
Supplement: Supplementary file 1 — supplemental material [file 41420_2024_2171_MOESM1_ESM.docx]

**Supplemental figure1**


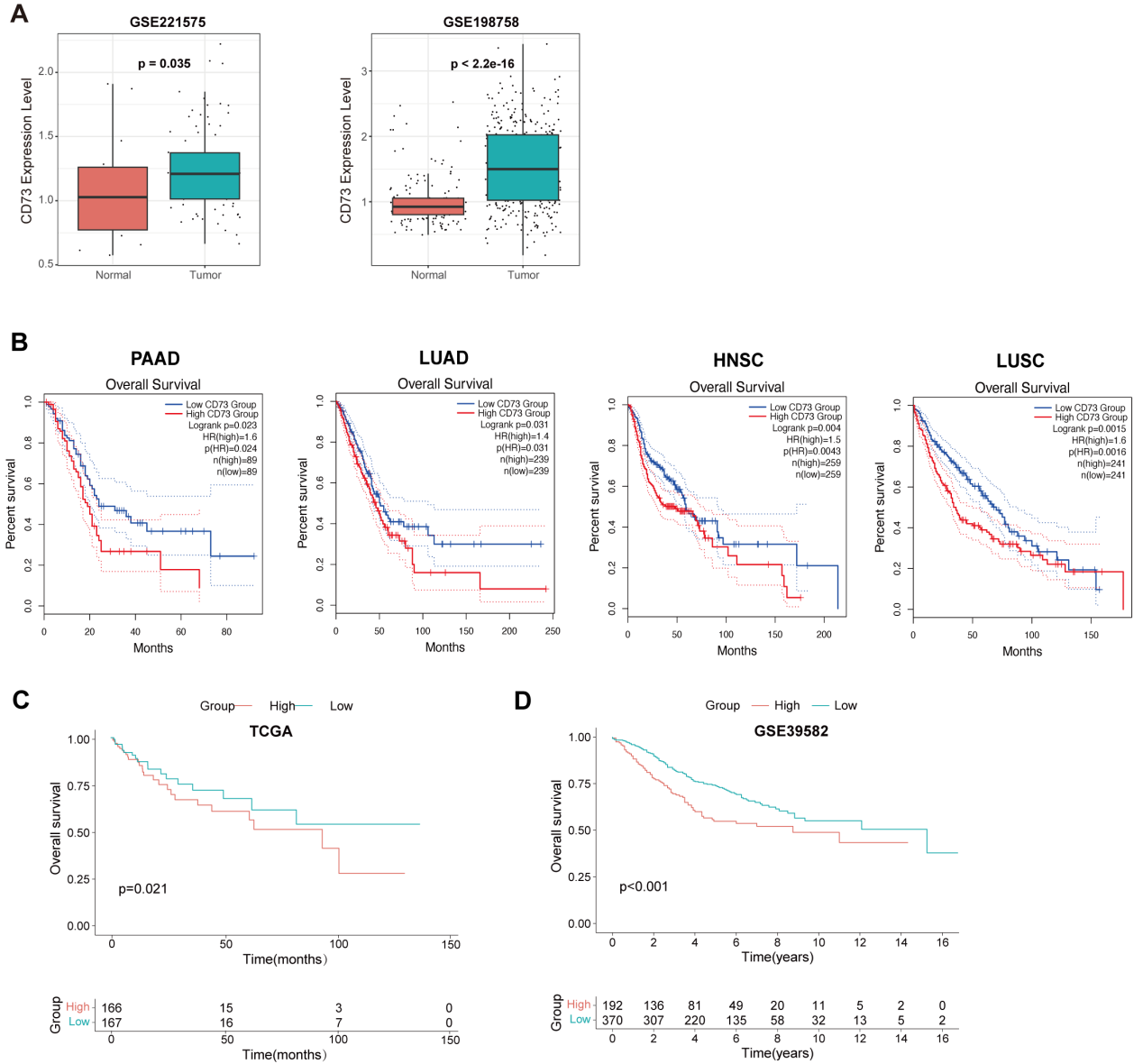


**Fig. S1. High CD73 expression is associated with poor prognosis in cancer patients.**

(A) The difference in CD73 expression in CRC tumors and normal adjacent tissues from GEO databases. (B)Survival curves for overall survival of the CD73 high and CD73 low groups in PAAD, LUAD, HNSC, and LUSC (B) patients from Gepia. (C, C) Survival curves for overall survival of the CD73 high and CD73 low groups in CCR patients from TCGA (C) and GEO (D) databases. PAAD, Pancreatic adenocarcinoma; LUAD, Lung adenocarcinoma; HNSC, Head and neck squamous cell carcinoma; LUSC, Lung squamous cell carcinoma; CRC, colorectal cancer.

**Supplemental figure 2**


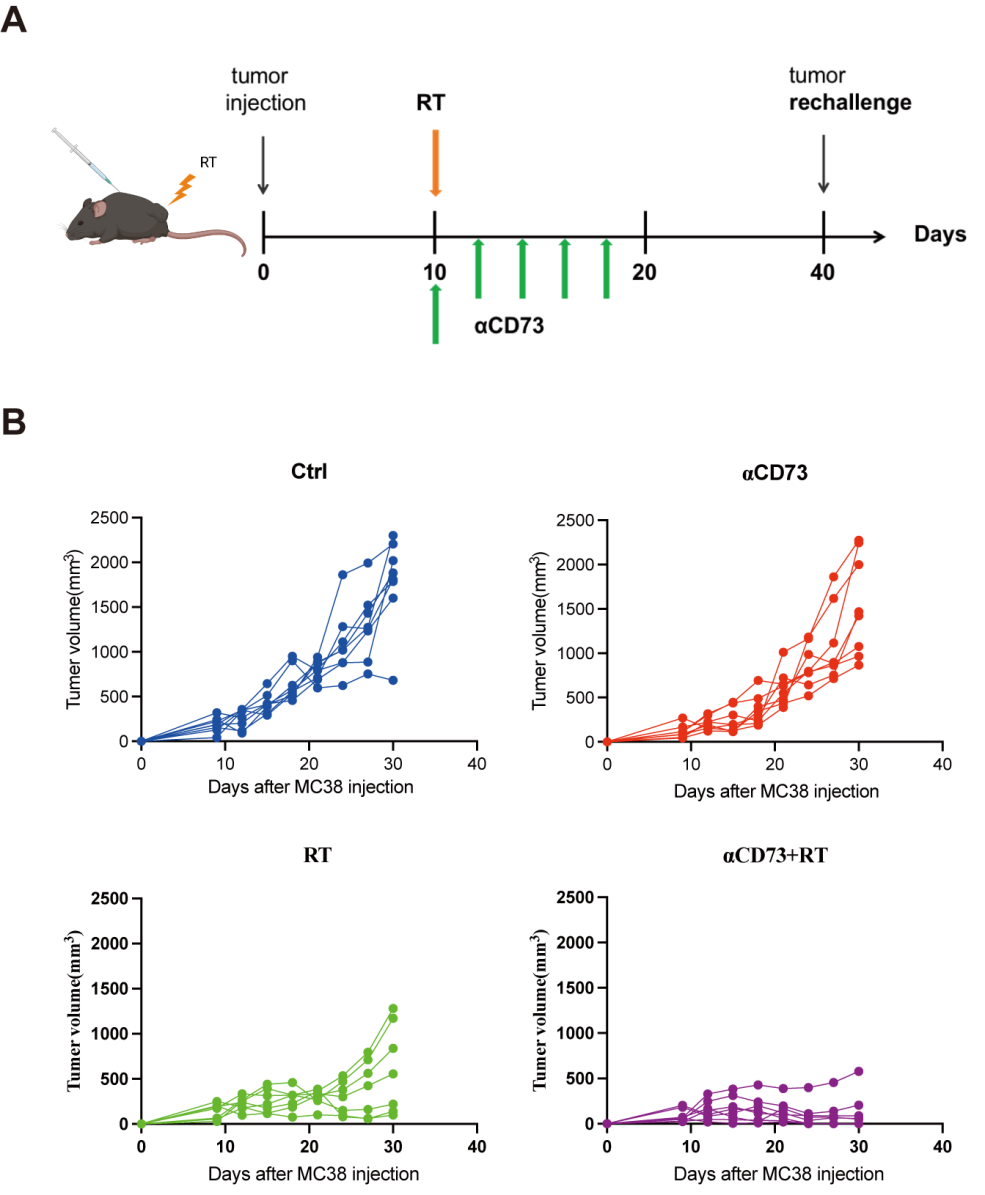


**Fig. S2. CD73 blockade combined with RT enhances anti-tumor response**

1. C57BL/6 mice were given through s.c. injection into the right flank with 1 × 10^6^ MC38 cells. Once tumor size achieved 100 mm^3^ (about 10 days after injection), a single dose of 8Gy RT was administered locally to mice. Simultaneously, αCD73 treatment was initiated, with five doses administered every other day. (B) Tumor growth curves of individual mice.

**Supplemental figure 3**


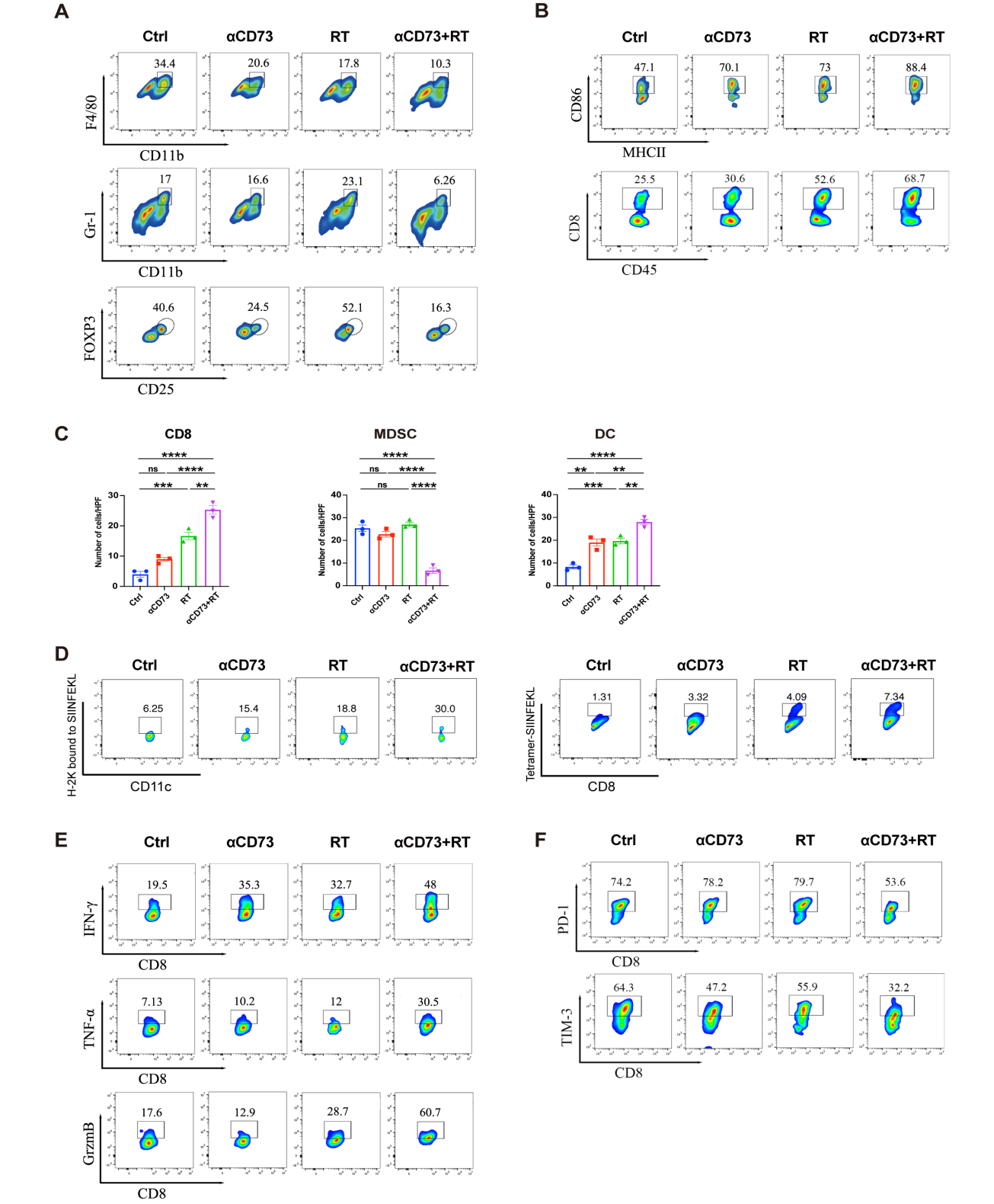


**Fig. S3. CD73 blockade enhanced RT-mediated antitumor immune effects and remodeled the TME**

(A, B) Representative images of flow cytometric analysis of TAMs (CD11b+F4/80+), and MDSCs (CD11b+Gr-1+) as a proportion of live CD45+ cells in the tumor, Tregs as a proportion of live CD4+ cells in the tumor (A). Mature DCs (CD86+) as a proportion of CD11b+CD11c+MHC-II+ cells in the tumor, and CD8+ T cells as a proportion of live CD45+ cells in the tumor (B). (C) Quantifications of CD8+, Gr1+, and CD86+ cells infiltration in tumors. (D) Representative images of flow cytometric analysis of H-2KbSIINFEKL+ cross-presenting DCs and H2-Kb - SIINFEKL-tetramer+ CD8+T cells in the tumor. (E, F) Representative images of flow cytometric analysis of IFN-γ+, TNF-α+, and GrzmB+cells as a proportion of CD8+ cells in the tumor (E), PD-1+ and TIM3+ cells as a proportion of CD8+ cells in the tumor (F).

**Supplemental figure 4**


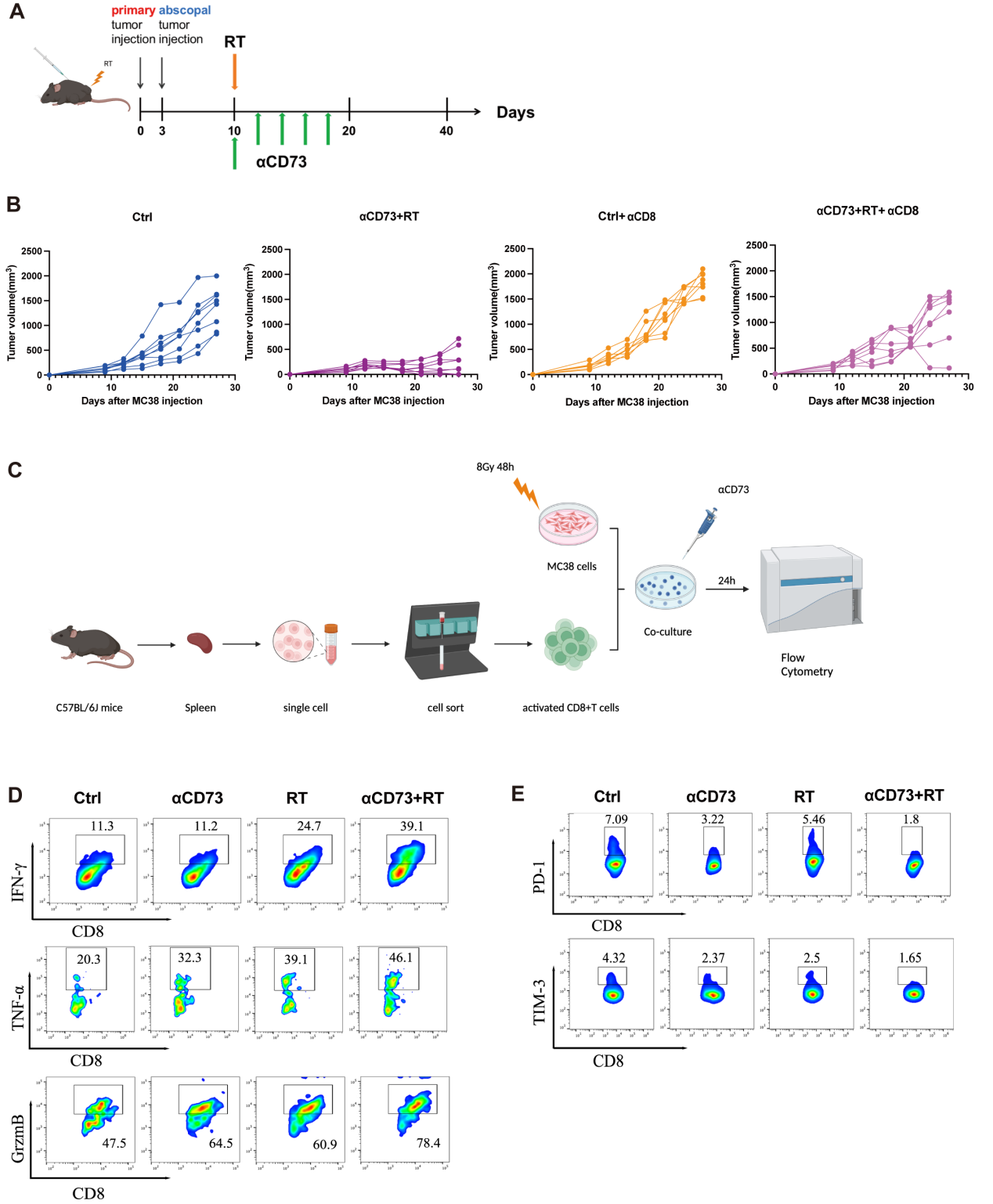


**Fig. S4. CD8 is indispensable in blocking CD73 to enhance RT-mediated immune response.**

(A) 1 × 106 MC38 cells were injected into the left flank of C57BL/6 mice three days after the first injection (1 × 106) into the right flank. For CT26 model, we utilized 2 × 105 CT26 cells injecting into BALB/c mice. The treatment schema was as described above. (B) In CD8+ T cells depletion experiment, the first anti-CD8 antibodies were administered three days prior to RT, with subsequent applications twice weekly throughout the course of treatment. Tumor volumes of individual mice in different groups are shown. (C) Splenic CD8+ T cells were isolated using magnetic bead isolation (Miltenyi Biotec) and pre-activated with Dynabeads mouse CD3/CD28 beads (IBA Life Sciences) for 48 hours. After 48 hours of pre-activation, they were co-cultured with MC38 cells treated ± 8 Gy RT, ± CD73i for 48 hours, followed by flow analysis 24 hours later. (D) Representative images of flow cytometric analysis of IFN-γ+, TNF-α+, and GrzmB+cells as a proportion of CD8+ cells. (E) Representative images of PD-1+ and TIM3+ cells as a proportion of CD8+ cells.

**Supplemental figure 5**

**
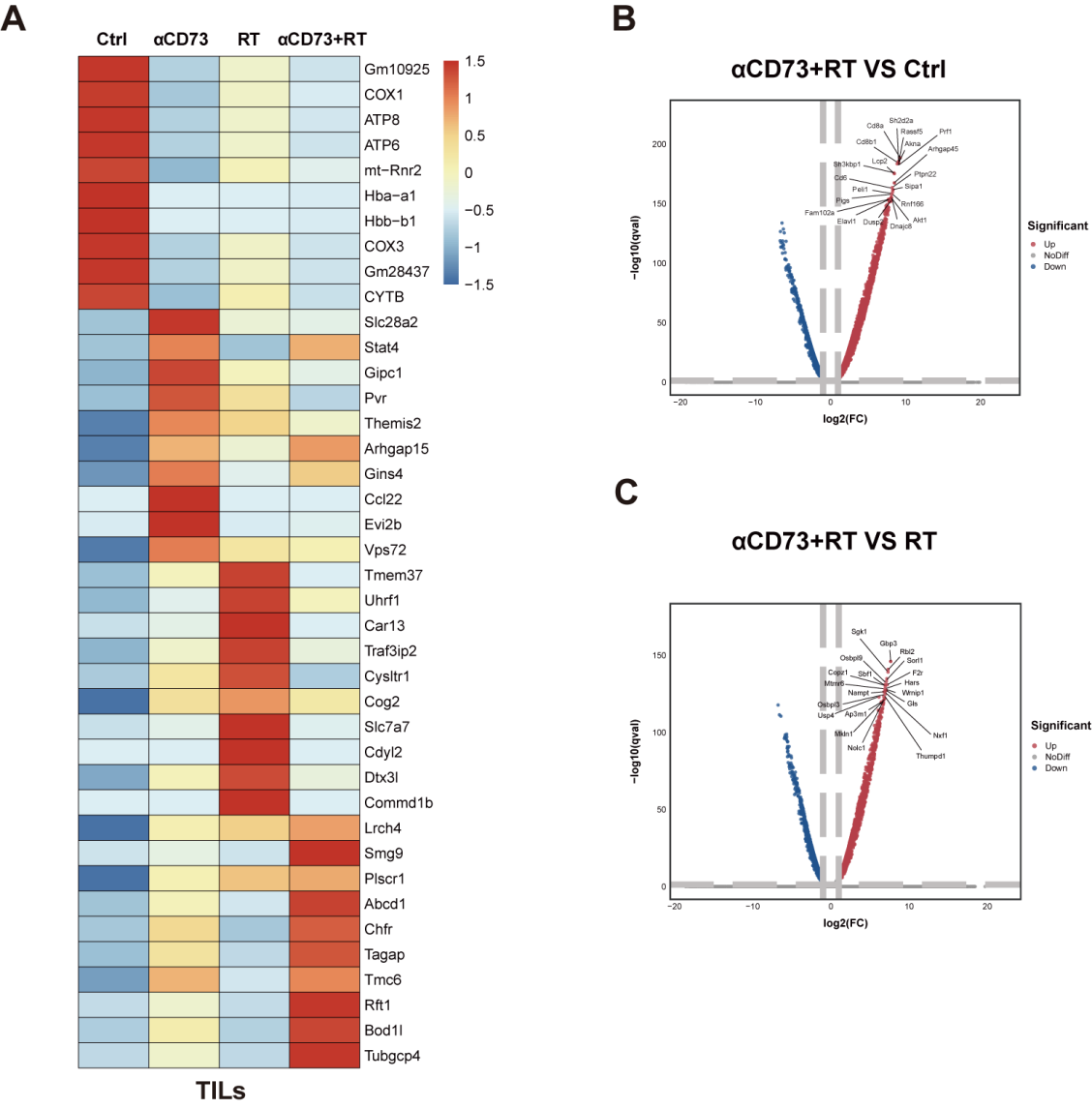
**

**Fig. S5. The combination of CD73 blockade and RT remodels the TME which relies on the STING pathway.**

1. Heat map demonstrating the top ten highly expressed genes in TILs isolated from tumors in control, αCD73, RT, and αCD73+RT groups. (B,C) The violin plot of the top ten up-regulated genes in the αCD73+RT group (αCD73+RT group vs control group) (B), and in the αCD73+RT group (αCD73+RT group vs αCD73 group) (C).

**Supplemental figure 6**


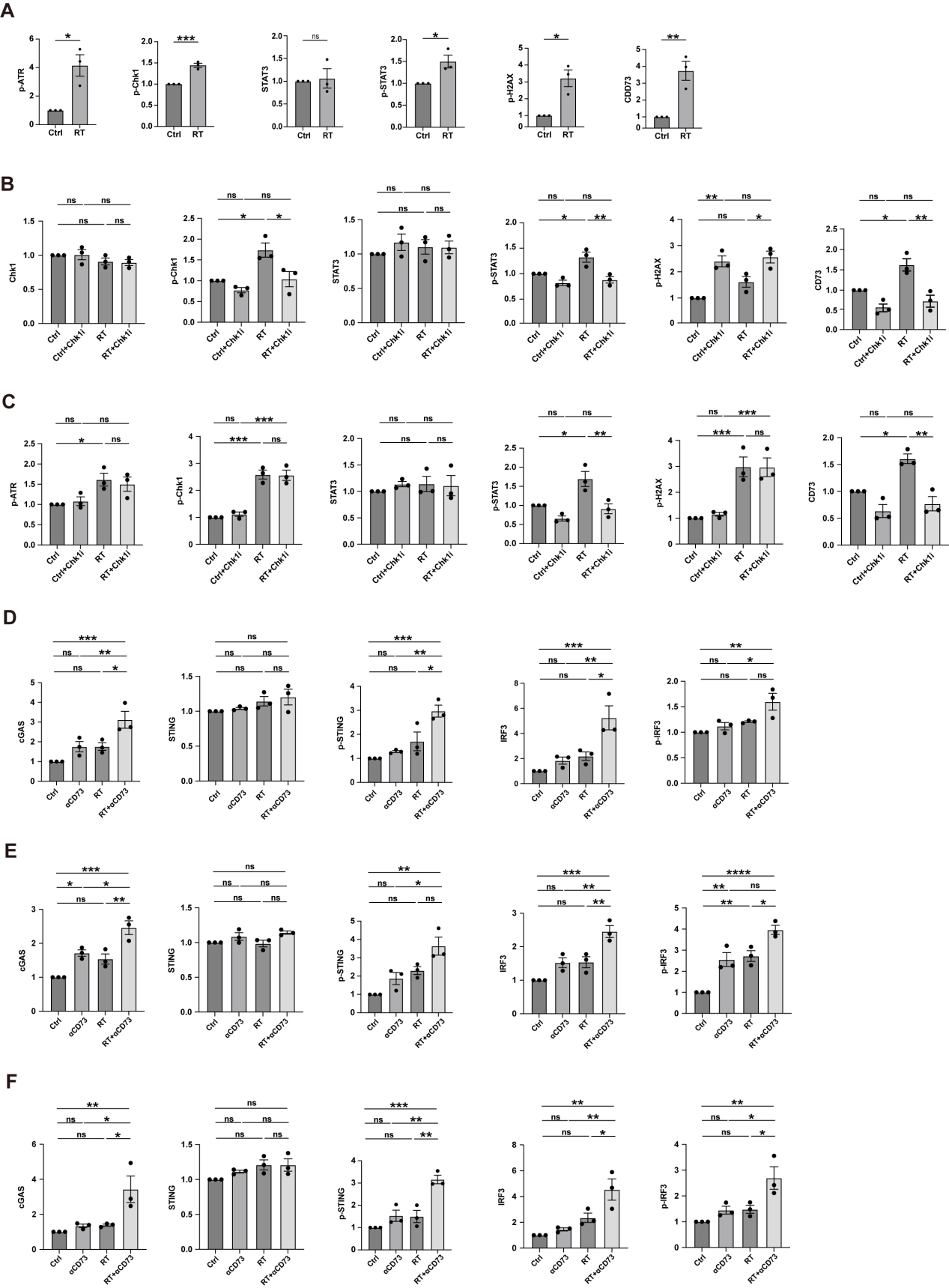


**Fig. S6. Images of the Western blot**

The ratio of the quantified values of the different treatment groups to the quantified values of the Ctrl group were analyzed to quantify the results for Western blots of Figure 2E (A), Figure 2F (B), Figure 2G (C), Figure 5E (C) and Figure 5F (E, F). DNA Damage Antibody Sampler Kit (catalog No. 9947, CST), Phospho-Chk1 (Ser296) Rabbit mAb (catalog No. 90178, CST), Chk1 Rabbit mAb (catalog No. 37010, CST), Mouse-Reactive STING Pathway Antibody Sample (catalog No. 16029, CST) and GAPDH Rabbit mAb (catalog No. 92310, CST) were used for immunoblotting. All primary antibodies were diluted at a ratio of 1:1000 and incubated overnight on a shaker at 4°C. Statistical variations were analyzed utilizing the unpaired t-test. Data are expressed as mean ± SEM (n = 3 per group).*P < 0.05; **P < 0.01; ***P < 0.001; ****P < 0.0001.

**Supplemental figure 7**

**
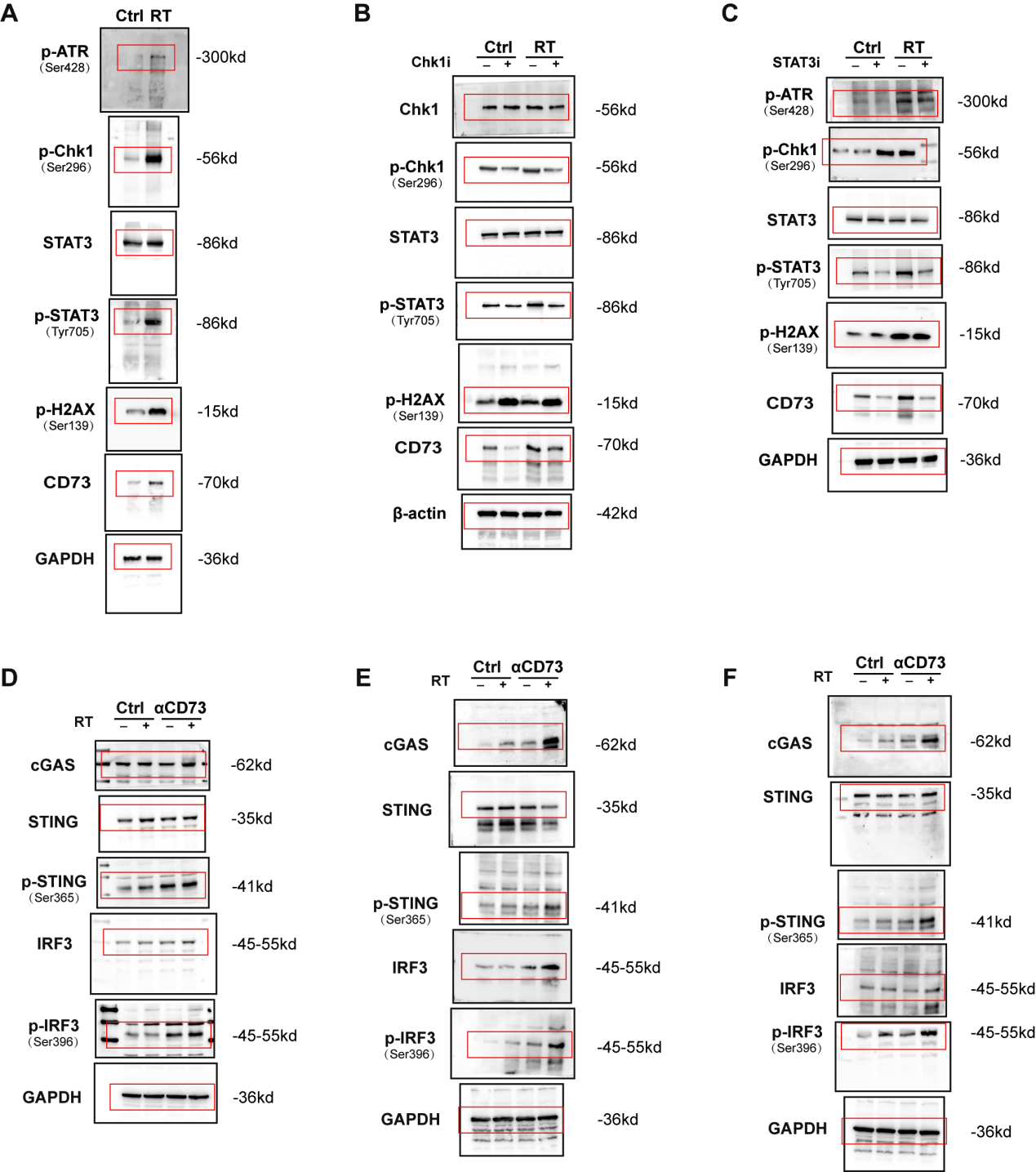
**

**Fig. S7. Images of the Western blot**

Original gels of the Western blots of Figure 2E (A), Figure 2F (B), Figure 2G (C), Figure 5E (D) and Figure 5F(E, F) are included. DNA Damage Antibody Sampler Kit (catalog No. 9947, CST), Mouse-Reactive STING Pathway Antibody Sample (catalog No. 16029, CST), Phospho-Chk1 (Ser296) Rabbit mAb (catalog No. 90178, CST), Chk1 Rabbit mAb (catalog No. 37010, CST), Phospho-Stat3 (Tyr705) Rabbit mAb (catalog No. 9145, CST)，Stat3 Rabbit mAb (catalog No. 30835, CST), β-Actin Antibody (catalog No. 4967, CST) and GAPDH Rabbit mAb (catalog No. 92310, CST) were used for immunoblotting. All primary antibodies were diluted at a ratio of 1:1000 and incubated overnight on a shaker at 4°C.

**Table S1. Details of Antibodies for Flow Cytometry**

| **Antibodies** | **Source** | **Identifier** |
| --- | --- | --- |
| Zombie UV™ Fixable Viability Kit | Biolegend | catalog No. 100752 |
| CD16/32 | Biolegend | clone 93, catalog No. 103132 |
| CD45- PerCP/Cy5.5 | Biolegend | clone 30-F11, catalog No. 103132 |
| CD45-APC/Cy7 | Biolegend | clone 30-F11, catalog No. 103116 |
| CD11b-FITC | Biolegend | clone M1/70, catalog No. 101206 |
| Gr-1- PerCP/Cy5.5 | Biolegend | clone RB6-8C5, catalog No. 108428 |
| F4/80- BV605 | Biolegend | clone BM8, catalog No. 123133 |
| CD11c- PE/Cy7 | Biolegend | clone N418, catalog No. 117318 |
| CD86-BV421 | Biolegend | clone GL-1, catalog No. 105032 |
| MHCII-PE/Cy7 | Biolegend | clone M5/114.15.2, catalog No. 107630 |
| CD8a-PE | Biolegend | clone 53-6.7, catalog No. 100708 |
| CD8-BV510 | Biolegend | clone 53-6.7, catalog No. 100752 |
| PD1-BV605 | Biolegend | clone 29F.1A12, catalog No.135219 |
| TIM-3-BV421 | Biolegend | clone RMT3-23, catalog No. 119723 |
| CD73-APC | Biolegend | clone TY/11.8, catalog No. 127209 |
| CD4- FITC | Biolegend | clone GK1.5, catalog No. 100406 |
| Mouse regulatory T cell staining kit | eBioscience | clone FJK-16s, catalog No. 88-8118 |
| TNF-α-BV421 | Biolegend | clone MP6-XT22, catalog No. 506328 |
| IFN-γ-PE | Biolegend | clone XMG1.2, catalog No.505808 |
| Granzyme B-APC | Biolegend | clone QA16A02, catalog No. 372203 |
| SIINFEKL-PE | ThermoFisher | clone eBio25-D1.16 (25-D1.16)，catalog No. 12-5743-82 |
| Tetramer-SIINFEKL-PE | MBL | TS-5001-1C |

**Table S2. Patient and treatment characteristics**

| **Variable** | **Category** | **n** | **Responder** | | ***P*** |
| --- | --- | --- | --- | --- | --- |
|  |  |  | **Good(n=12)** | **Poor(n=13)** |  |
| **Age** | >=50 | 21 | 10 | 11 | 0.930 |
|  | <50 | 4 | 2 | 2 |  |
| **Gender** | Male | 17 | 7 | 10 | 0.319 |
|  | Female | 8 | 5 | 3 |  |
| **cT stage** | T3 | 20 | 9 | 11 | 0.548 |
|  | T4 | 5 | 3 | 2 |  |
| **cN stage** | N0 | 11 | 5 | 6 | 0.371 |
|  | N1 | 9 | 6 | 3 |  |
|  | N2 | 5 | 1 | 4 |  |
| **cM stage** | M0 | 25 | 12 | 13 |  |
| **cTNM stage** | II | 11 | 5 | 6 | 0.302 |
|  | III | 14 | 7 | 7 |  |
| **Differentiation** | Well | 3 | 1 | 2 | 0.152 |
|  | Moderate | 19 | 11 | 8 |  |
|  | Poor | 3 | 0 | 3 |  |
| **TRG** | Grade 2 | 4 | 4 | 0 | **-** |
|  | Grade 3 | 6 | 6 | 0 |  |
|  | Grade 4 | 9 | 2 | 7 |  |
|  | Grade 5 | 6 | 0 | 6 |  |
| **ypT stage** | T1 | 1 | 1 | 0 | 0.511 |
|  | T2 | 3 | 1 | 2 |  |
|  | T3 | 21 | 10 | 11 |  |
| **ypN stage** | N0 | 14 | 7 | 7 | 0.464 |
|  | N1 | 4 | 3 | 1 |  |
|  | N2 | 7 | 2 | 5 |  |
| **ypM stage** | M0 | 25 | 12 | 13 |  |
| **ypTNM stage** | I | 2 | 2 | 0 | 0.128 |
|  | II | 12 | 5 | 7 |  |
|  | III | 11 | 5 | 6 |  |

All patients received a combination of short-course radiotherapy followed by sequential chemotherapy. The chemotherapy regimen employed was CAPOX. The radiotherapy protocol consisted of 5 Gy*5, with the entire radiotherapy treatment completed within one week.

Tumor regression grade (TRG) was defined according to the Mandard system: grade 2, rare residual cancer cells; grade 3, fibrosis outgrowing residual cancer; grade 4, residual cancer outgrowing ﬁbrosis; grade 5, absence of regressive changes. Results for continuous variables are expressed as mean ± standard errors.
