# Supplementary figures and images for: Blockade of CD73 potentiates radiotherapy antitumor immunity and abscopal effects via STING pathway
Source: Cell Death Discov. 2024 Sep 16;10:404. doi: 10.1038/s41420-024-02171-4 (PMC11405876; doi:10.1038/s41420-024-02171-4)

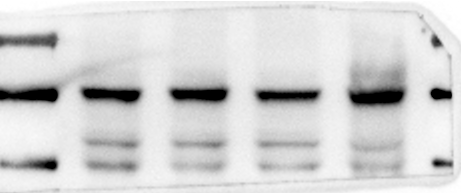

Supplement: Supplementary file 2 — Western blot [file 41420_2024_2171_MOESM2_ESM.zip › image/Fig5E/cGAS.tif]

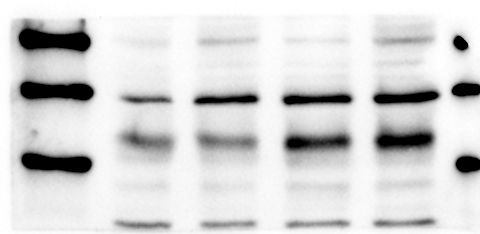

Supplement: Supplementary file 2 — Western blot [file 41420_2024_2171_MOESM2_ESM.zip › image/Fig5E/p-IRF3.tif]

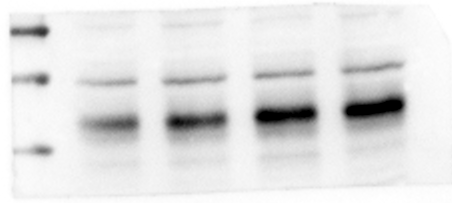

Supplement: Supplementary file 2 — Western blot [file 41420_2024_2171_MOESM2_ESM.zip › image/Fig5E/p-STING.tif]

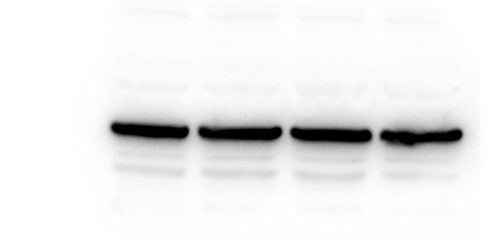

Supplement: Supplementary file 2 — Western blot [file 41420_2024_2171_MOESM2_ESM.zip › image/Fig5E/GAPDH.tif]

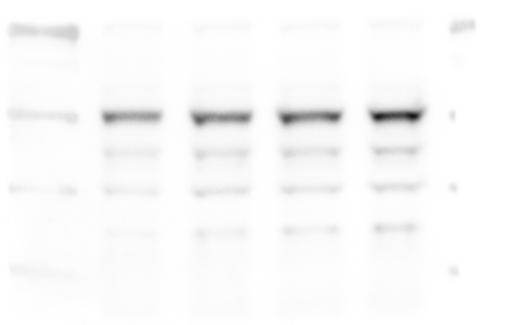

Supplement: Supplementary file 2 — Western blot [file 41420_2024_2171_MOESM2_ESM.zip › image/Fig5E/IRF3.tif]

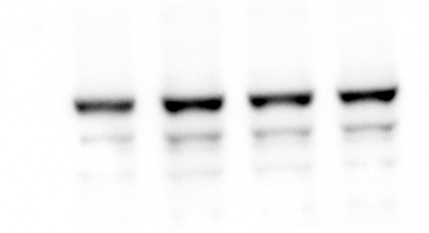

Supplement: Supplementary file 2 — Western blot [file 41420_2024_2171_MOESM2_ESM.zip › image/Fig5E/STING.tif]

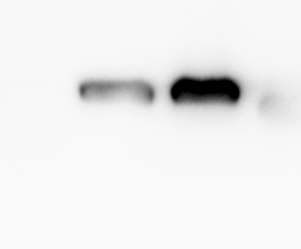

Supplement: Supplementary file 2 — Western blot [file 41420_2024_2171_MOESM2_ESM.zip › image/Fig2E/P-H2AX.tif]

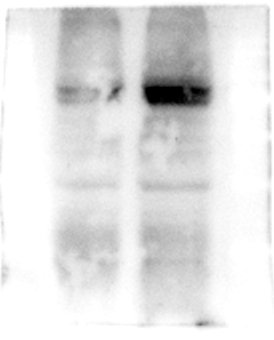

Supplement: Supplementary file 2 — Western blot [file 41420_2024_2171_MOESM2_ESM.zip › image/Fig2E/p-STAT3.tif]

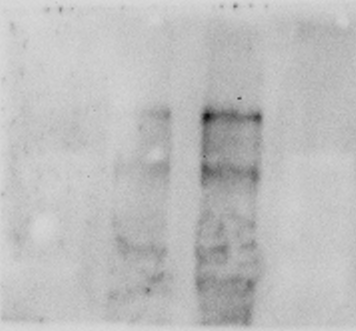

Supplement: Supplementary file 2 — Western blot [file 41420_2024_2171_MOESM2_ESM.zip › image/Fig2E/p-ATR.tif]

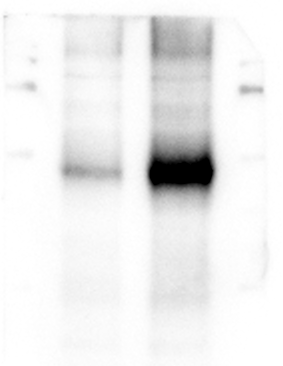

Supplement: Supplementary file 2 — Western blot [file 41420_2024_2171_MOESM2_ESM.zip › image/Fig2E/p-Chk1.tif]

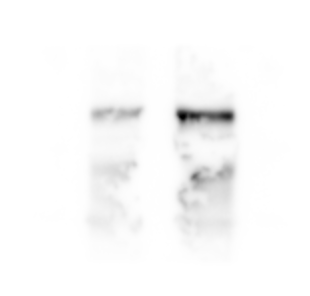

Supplement: Supplementary file 2 — Western blot [file 41420_2024_2171_MOESM2_ESM.zip › image/Fig2E/CD73.tif]

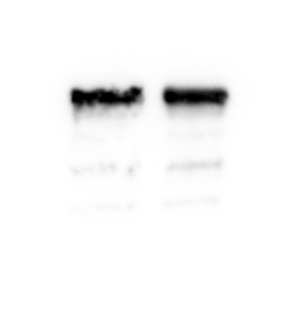

Supplement: Supplementary file 2 — Western blot [file 41420_2024_2171_MOESM2_ESM.zip › image/Fig2E/GAPDH.tif]

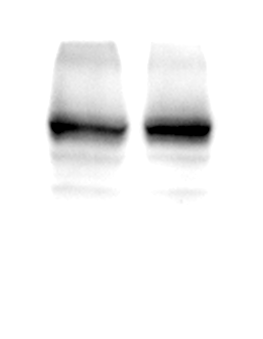

Supplement: Supplementary file 2 — Western blot [file 41420_2024_2171_MOESM2_ESM.zip › image/Fig2E/STAT3.tif]

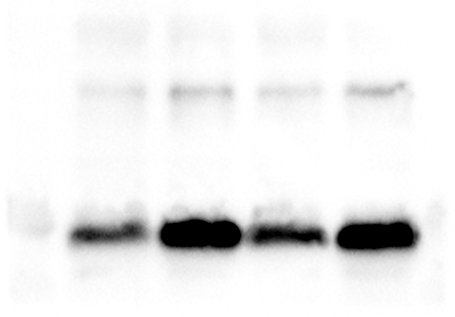

Supplement: Supplementary file 2 — Western blot [file 41420_2024_2171_MOESM2_ESM.zip › image/Fig2F/P-H2AX.tif]

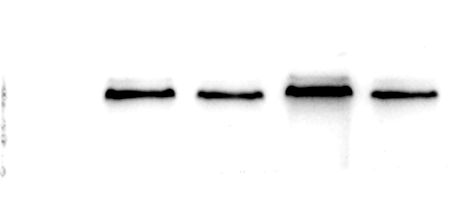

Supplement: Supplementary file 2 — Western blot [file 41420_2024_2171_MOESM2_ESM.zip › image/Fig2F/p-STAT3.tif]

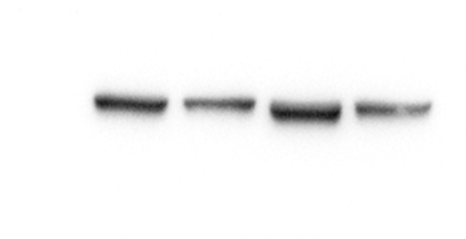

Supplement: Supplementary file 2 — Western blot [file 41420_2024_2171_MOESM2_ESM.zip › image/Fig2F/p-Chk1.tif]

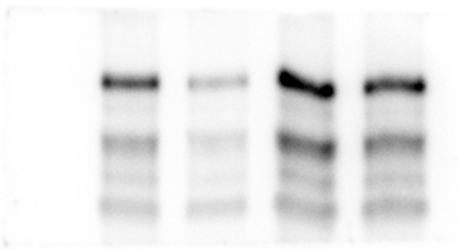

Supplement: Supplementary file 2 — Western blot [file 41420_2024_2171_MOESM2_ESM.zip › image/Fig2F/CD73.tif]

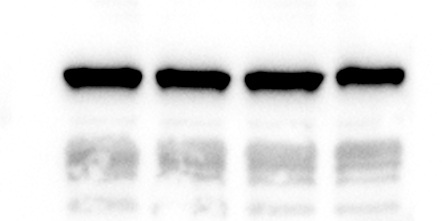

Supplement: Supplementary file 2 — Western blot [file 41420_2024_2171_MOESM2_ESM.zip › image/Fig2F/╬▓-actin.tif]

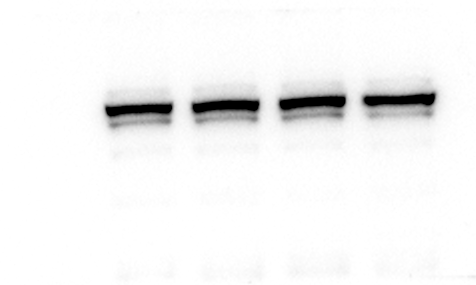

Supplement: Supplementary file 2 — Western blot [file 41420_2024_2171_MOESM2_ESM.zip › image/Fig2F/STAT3.tif]

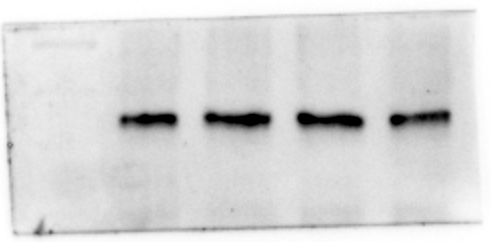

Supplement: Supplementary file 2 — Western blot [file 41420_2024_2171_MOESM2_ESM.zip › image/Fig2F/Chk1.tif]

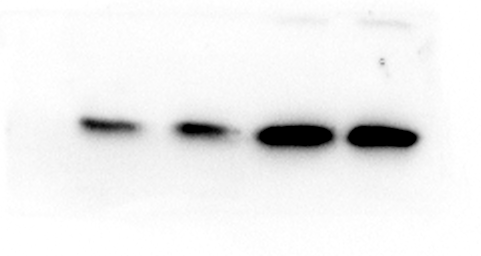

Supplement: Supplementary file 2 — Western blot [file 41420_2024_2171_MOESM2_ESM.zip › image/Fig2G/p-H2AX.tif]

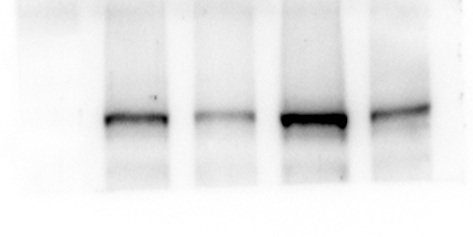

Supplement: Supplementary file 2 — Western blot [file 41420_2024_2171_MOESM2_ESM.zip › image/Fig2G/p-STAT3.tif]

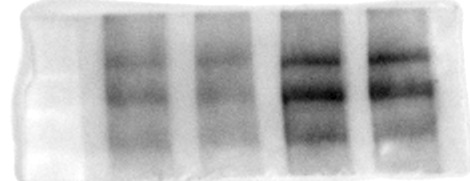

Supplement: Supplementary file 2 — Western blot [file 41420_2024_2171_MOESM2_ESM.zip › image/Fig2G/p-ATR.tif]

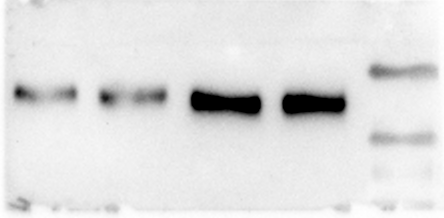

Supplement: Supplementary file 2 — Western blot [file 41420_2024_2171_MOESM2_ESM.zip › image/Fig2G/p-Chk1.tif]

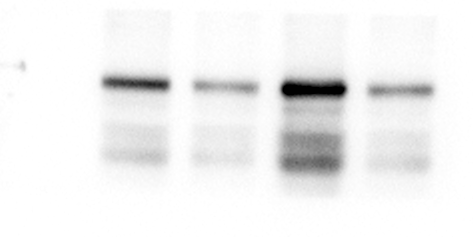

Supplement: Supplementary file 2 — Western blot [file 41420_2024_2171_MOESM2_ESM.zip › image/Fig2G/CD73.tif]

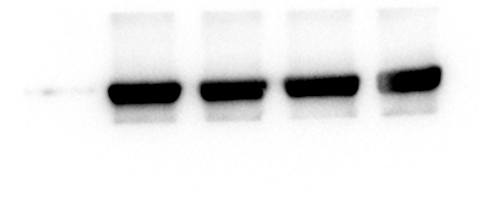

Supplement: Supplementary file 2 — Western blot [file 41420_2024_2171_MOESM2_ESM.zip › image/Fig2G/GAPDH.tif]

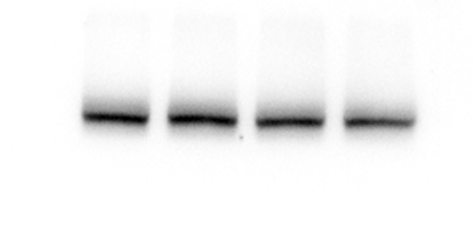

Supplement: Supplementary file 2 — Western blot [file 41420_2024_2171_MOESM2_ESM.zip › image/Fig2G/STAT3.tif]

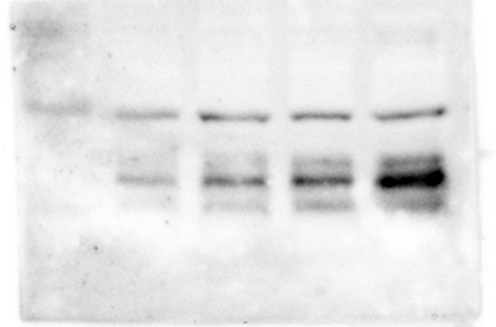

Supplement: Supplementary file 2 — Western blot [file 41420_2024_2171_MOESM2_ESM.zip › image/Fig5F/Secondery/cGAS.tif]

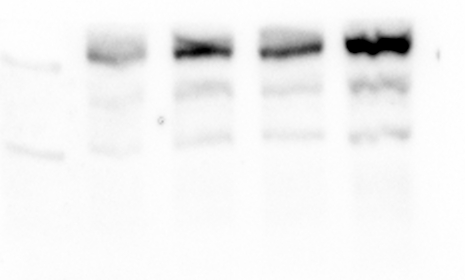

Supplement: Supplementary file 2 — Western blot [file 41420_2024_2171_MOESM2_ESM.zip › image/Fig5F/Secondery/p-IRF3.tif]

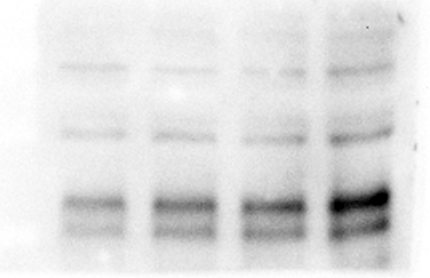

Supplement: Supplementary file 2 — Western blot [file 41420_2024_2171_MOESM2_ESM.zip › image/Fig5F/Secondery/p-STING.tif]

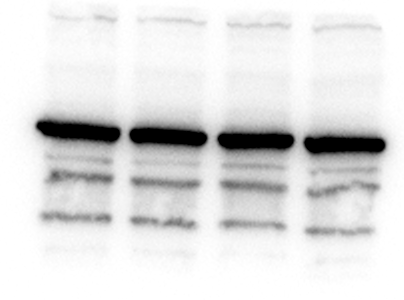

Supplement: Supplementary file 2 — Western blot [file 41420_2024_2171_MOESM2_ESM.zip › image/Fig5F/Secondery/GAPDH.tif]

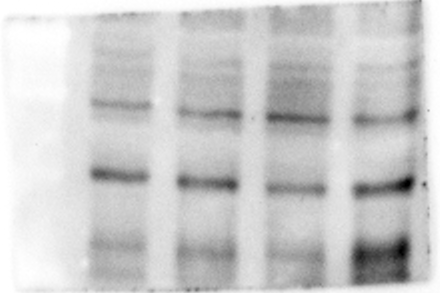

Supplement: Supplementary file 2 — Western blot [file 41420_2024_2171_MOESM2_ESM.zip › image/Fig5F/Secondery/IRF3.tif]

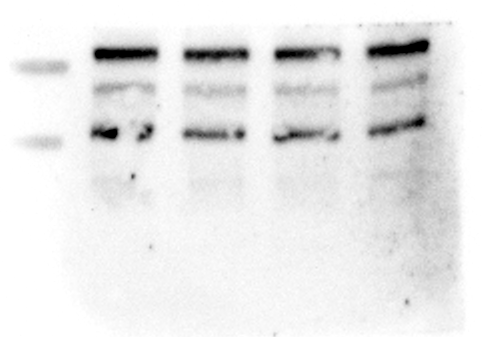

Supplement: Supplementary file 2 — Western blot [file 41420_2024_2171_MOESM2_ESM.zip › image/Fig5F/Secondery/STING.tif]

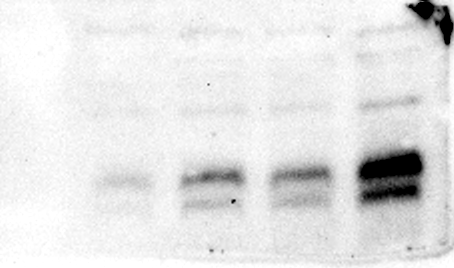

Supplement: Supplementary file 2 — Western blot [file 41420_2024_2171_MOESM2_ESM.zip › image/Fig5F/Primary/cGAS.tif]

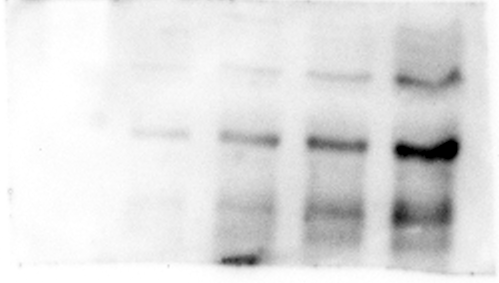

Supplement: Supplementary file 2 — Western blot [file 41420_2024_2171_MOESM2_ESM.zip › image/Fig5F/Primary/p-IRF3.tif]

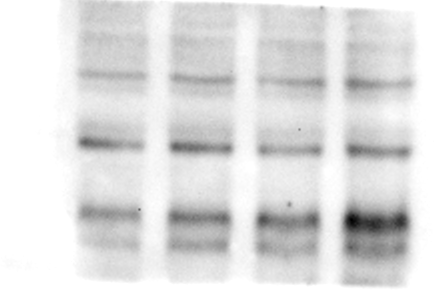

Supplement: Supplementary file 2 — Western blot [file 41420_2024_2171_MOESM2_ESM.zip › image/Fig5F/Primary/p-STING.tif]

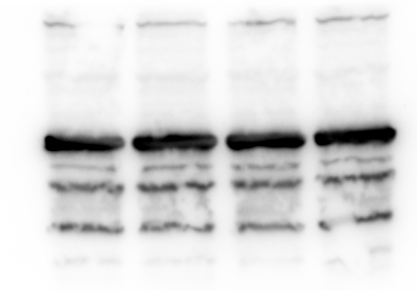

Supplement: Supplementary file 2 — Western blot [file 41420_2024_2171_MOESM2_ESM.zip › image/Fig5F/Primary/GAPDH.tif]

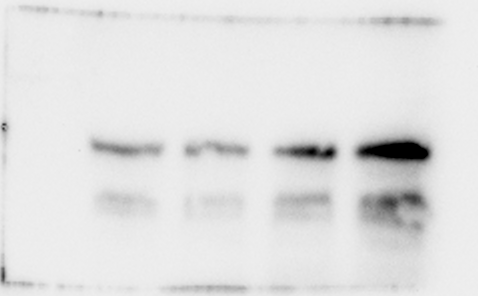

Supplement: Supplementary file 2 — Western blot [file 41420_2024_2171_MOESM2_ESM.zip › image/Fig5F/Primary/IRF3.tif]

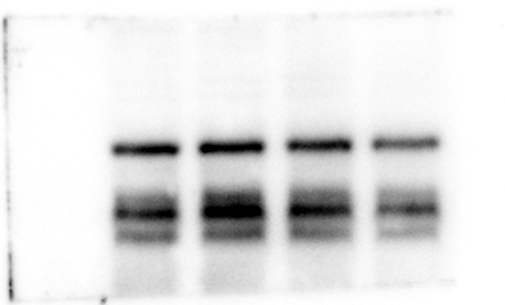

Supplement: Supplementary file 2 — Western blot [file 41420_2024_2171_MOESM2_ESM.zip › image/Fig5F/Primary/STING.tif]
